# Nanoparticle Drug Delivery Systems for Atherosclerosis: Precision Targeting, Inflammatory Modulation, and Plaque Stabilization

**DOI:** 10.1002/advs.202504990

**Published:** 2025-08-28

**Authors:** Yunqi Zhang, Feifei Li, Libo Liu, Ziyu An, Hanzhong Luo, Huan Zhang, Rui Ma, Ruoxi Zhang, Qiong Dai, Mingduo Zhang, Jinfan Tian, Chaoyong Liu, Xiantao Song, Yunfeng Lu

**Affiliations:** ^1^ Department of Cardiology Beijing Anzhen Hospital Capital Medical University Beijing 100029 P. R. China; ^2^ State Key Laboratory of Organic‐Inorganic Composites Beijing Advanced Innovation Center for Soft Matter Science and Engineering College of Life Science and Technology Beijing University of Chemical Technology Beijing 100029 P. R. China

**Keywords:** atherosclerosis, drug delivery, nanomedicine, nanoparticles

## Abstract

Atherosclerosis remains a leading contributor to cardiovascular morbidity and mortality, presenting considerable therapeutic challenges due to its multifaceted and complex pathogenesis. This review systematically explores the pathophysiological mechanisms underlying atherosclerosis and examines recent advances in nanoparticle drug delivery systems (NDDSs) tailored for atherosclerosis treatment. NDDS has many advantages in this setting. It can precisely modulate the cellular activities critical to atherosclerotic progression, and by using targeted delivery strategies directed at lesion‐specific biomarkers and incorporating stimulus‐responsive drug release mechanisms responsive to the plaque microenvironments, it can significantly increase therapeutic efficacy while minimizing adverse systemic and off‐target effects. Thus, NDDS effectively addresses the limitations of traditional pharmacological treatments, enabling personalized interventions that can attenuate disease progression, stabilize vulnerable plaques, and facilitate plaque regression. This review highlights the transformative possibilities of NDDSs, emphasizing their potential to redefine current therapeutic paradigms and improve patient outcomes in atherosclerosis.

## Graphical abstract

This review first summarizes the complex pathogenesis of atherosclerosis and outlines the limitations of conventional therapies. It then explores innovative nanoparticle drug delivery system (NDDS) developments, detailing their precision targeting strategies, responsiveness to plaque‐specific stimuli, modulation of key pathological processes, and potential for enhancing therapeutic efficacy, plaque stabilization, and regression, ultimately transforming the management of atherosclerosis.

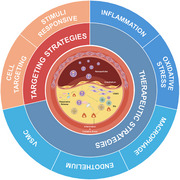

## 1 Introduction

Atherosclerosis is the most common cardiovascular disease. It is characterized by lipid accumulation, chronic inflammation, and plaque formation within the intimal layer of medium‐and large‐sized arteries. Atherosclerosis is a chronic and insidious process, and can remain asymptomatic for decades before having serious health consequences. As plaques develop, they gradually narrow the space inside the arterial lumen, impairing blood flow and disrupting the supply of oxygen and nutrients to organs and tissues. These ischemic insults can manifest in various ways within affected vascular territories, including as ischemic heart disease, ischemic strokes, or peripheral arterial disease. There is a substantial global burden of atherosclerosis, particularly in low‐and middle‐income countries. According to the 2021 Global Burden of Disease Study, ischemic heart disease and ischemic stroke accounted for 188.361 and 70.358 million disability‐adjusted life years, respectively, with mortality figures reaching 8.992 and 3.592 million, respectively. These alarming statistics underscore the need for more effective preventive approaches and treatments for atherosclerosis and its downstream clinical consequences.

The pathogenesis of atherosclerosis is complex. It involves endothelial dysfunction, immune cell infiltration, foam cell formation, vascular smooth muscle cell (VSMC) phenotype switching, and oxidative stress. Current therapeutic approaches include risk factor modification and medical and surgical treatments. Lipid‐lowering therapies, including statins, have significantly reduced cardiovascular morbidity and mortality in patients with atherosclerosis. However, a considerable residual risk remains, with about 2/3 of these patients experiencing recurrent cardiovascular events despite achieving their target low‐density lipoprotein cholesterol (LDL‐C) levels.^[^ ^1^ ^]^ Current surgical interventions, including percutaneous coronary intervention and coronary artery bypass grafting, provide symptomatic relief but do not address the underlying inflammatory or metabolic processes, which makes restenosis and long‐term complications fairly common. Additionally, patients undergoing these procedures typically require lifelong treatment with anticoagulants, antiplatelets, and lipid‐lowering agents to mitigate recurrence and thrombotic risk. Given these challenges, there is a growing demand for more precise, more targeted, and less invasive treatments that could effectively modulate the disease mechanisms underlying atherosclerosis.

Over the past several years, nanoparticle drug delivery systems (NDDSs) have emerged as a transformative method to treat atherosclerosis. They are advantageous compared to conventional therapies because they allow for site‐specific drug delivery and minimize systemic toxicity. NDDS can be designed to selectively accumulate within atherosclerotic plaques via receptor‐mediated targeting or microenvironment‐responsive mechanisms. These designs both enhance drug efficacy and reduce off‐target effects. NDDS can also facilitate the combined delivery of multiple therapeutic agents, allowing for simultaneous targeting of several pathologic processes (e.g., inflammation, oxidative stress, cholesterol metabolism, and immune responses). In addition to serving as passive carriers, some nanomaterials also have intrinsic therapeutic properties, further expanding their therapeutic potential in atherosclerosis.

Here, we aim to provide a comprehensive and up‐to‐date analysis of NDDS for atherosclerosis treatment. We will discuss the latest advances in nanoparticle formulations, targeting mechanisms, and controlled‐release strategies, as well as preclinical and clinical studies focused on the efficacy of NDDS. By integrating the most recent advances in NDDS and a detailed overview of cardiovascular pathophysiology, we aim to highlight NDDSs’ potential to transform the provision of personalized, site‐specific, and multimodal therapy for atherosclerosis. Understanding these innovations will aid in the development of next‐generation therapies for this devastating cardiovascular disease.

## 2 Pathophysiological Basis of Atherosclerosis

**Figure 1:**
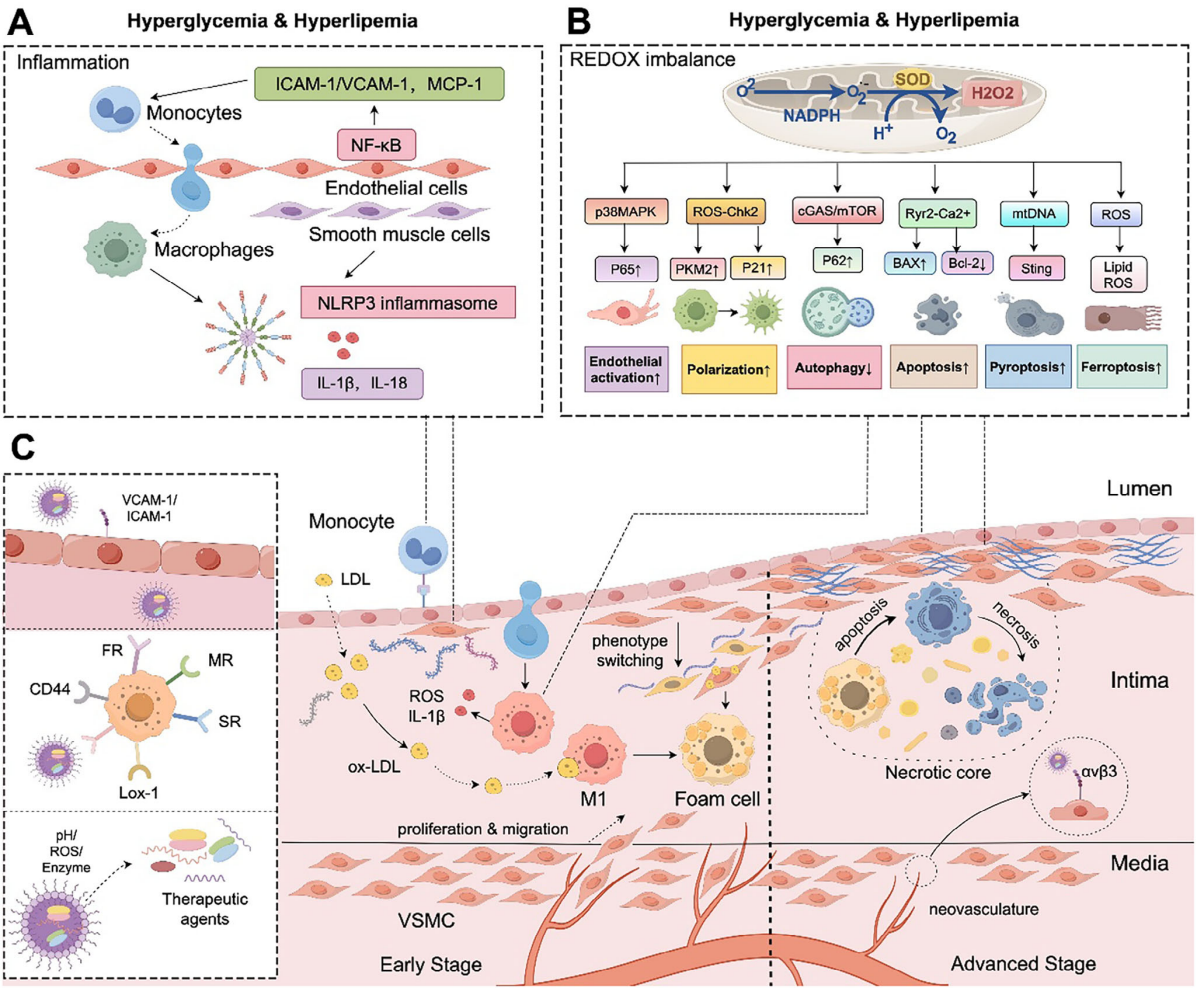
Major pathologic processes underlying atherosclerosis and relevant nanoparticle targeting mechanisms. Atherosclerosis is driven by a pathologic interplay between endothelial cells, macrophages, and vascular smooth muscle cells (VSMCs) and is also informed by inflammation and oxidative stress. A comprehensive understanding of these mechanisms is crucial to the development of targeted, efficient therapies. Hyperglycemia and hyperlipidemia exacerbate atherosclerosis via several convergent mechanisms. A) These metabolic disturbances activate the NF‐κB signaling pathway in endothelial cells, upregulating adhesion molecules and monocyte chemoattractant protein‐1 (MCP‐1) and promoting monocyte adhesion and transendothelial migration. Simultaneously, hyperglycemia and hyperlipidemia activate nucleotide‐binding oligomerization domain‐like receptor protein 3 (NLRP3) inflammasomes, leading to the maturation of proinflammatory cytokines [e.g., interleukin‐1β (IL‐1β) and IL‐18] and increasing vascular inflammation within atherosclerotic lesions. B) By inducing redox imbalance, hyperglycemia and hyperlipidemia can also influence endothelial cell activation, macrophage polarization, autophagy, and programmed cell death, all of which contribute to plaque progression. C) nanoparticles can be designed to selectively accumulate within atherosclerotic plaques, either via specific ligand–receptor interactions or via the release of therapeutic agents only in response to unique lesion microenvironments. This highly targeted approach reduces nonspecific distribution to healthy tissues and minimizes adverse effects.

To fully explore the mechanisms underlying nanoparticle‐based therapy for atherosclerosis, we must first gain a deep understanding of the processes involved in the onset and progression of atherosclerosis. Atherosclerosis is driven by a dynamic, pathologic interplay between endothelial cells, macrophages, and VSMCs. These interactions are shaped by inflammation and oxidative stress and drive both disease initiation and progression (**Figure** **1**).

**Figure 2:**
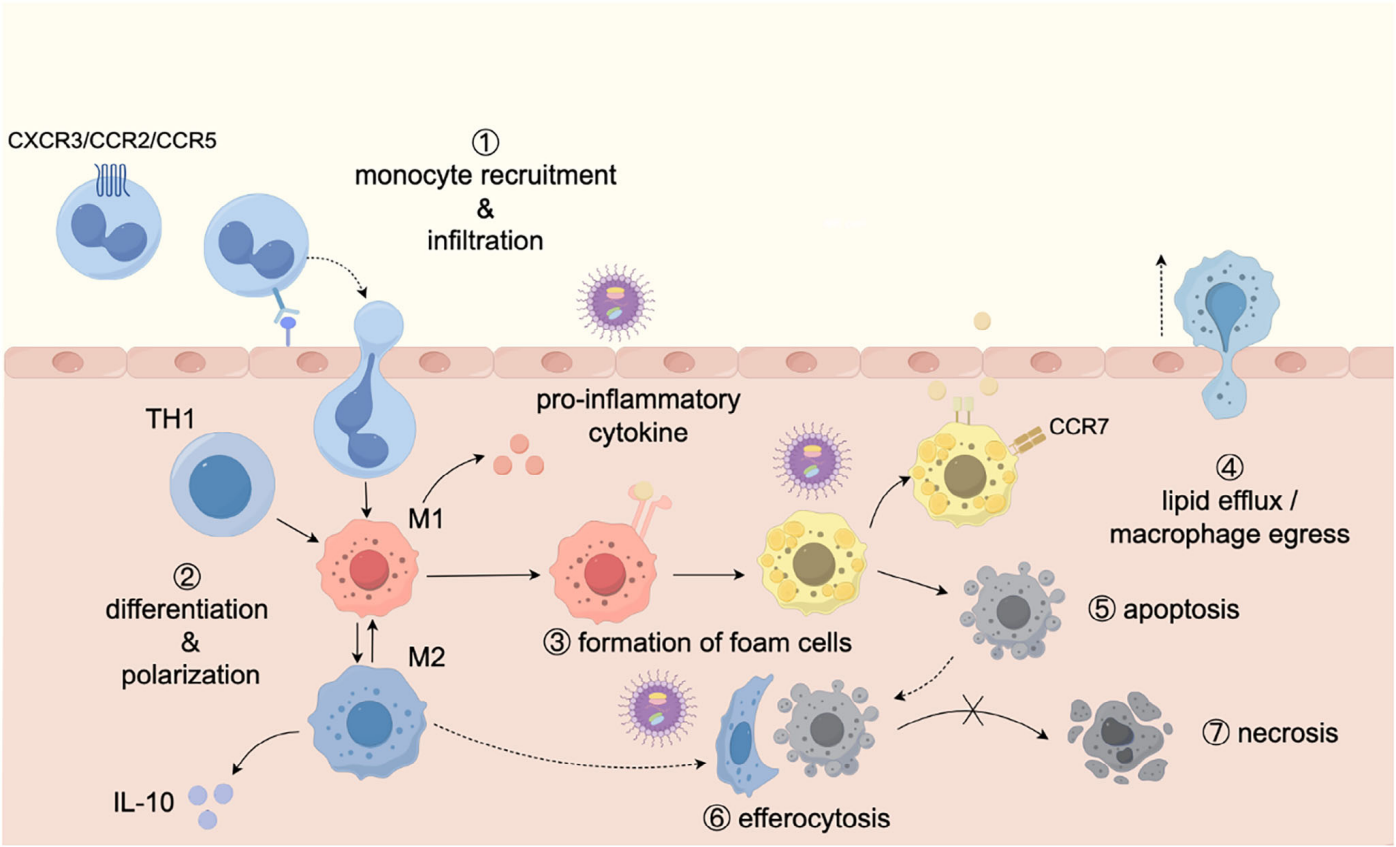
Nanoparticle modulation of macrophage behavior in atherosclerotic plaques. Monocytes and macrophages play a crucial role in the onset and progression of atherosclerosis. ① Initially, monocytes are recruited to and infiltrate the intima under the orchestrated action of chemokines and endothelial adhesion molecules. ② Subsequently, influenced by growth factors and cytokines, these monocytes proliferate and differentiate into distinct macrophage phenotypes. ③ Among these phenotypes, classically activated M1 macrophages drive atherogenesis by engulfing lipoproteins retained beneath the endothelium and transforming into lipid‐laden “foam cells.” ④ In the early stages of atherosclerosis, lesions may regress via mechanisms such as reverse cholesterol transport (RCT) and reverse macrophage migration. ⑤ However, prolonged lipid accumulation within macrophages can eventually trigger apoptosis. ⑥ Efficient clearance of these apoptotic cells by alternatively activated M2 macrophages is essential for inflammatory resolution, as M2 macrophages release anti‐inflammatory mediators. ⑦ Conversely, if these apoptotic cells evade phagocytosis, they undergo secondary necrosis, leading to the formation of necrotic lipid cores and driving plaque progression. Nanoparticle drug delivery system (NDDS) offers a promising avenue to modulate numerous macrophage behaviors, including infiltration, proliferation, polarization, lipid handling, and phagocytosis. By targeting these processes, NDDS could mitigate atherosclerotic progression and promote plaque stabilization.

### 2.1 Key Cellular Players in Lesion Development

#### 2.1.1 Endothelial Dysfunction

The injury‐response hypothesis is the most widely accepted theory about the pathogenesis of atherosclerosis. This hypothesis posits that atherosclerosis is initially caused by dysfunction or injury to endothelial cells, which normally serve as a barrier between the vascular lumen and the surrounding tissues.^[^ ^2^ ^]^ In addition to their structural role, endothelial cells also regulate vascular tone, coagulatory balance, and inflammatory responses by secreting select bioactive molecules. Endothelial dysfunction is marked by an imbalance between vasodilatory and vasoconstrictive factors, which leads to impaired vascular relaxation and a cascade of disturbances in endothelial homeostasis.

In physiologic conditions, endothelial cells produce nitric oxide (NO) from arginine via the enzyme endothelial NO synthase (eNOS). NO is a key regulator of vascular homeostasis. It functions as a potent vasodilator and has anti‐inflammatory, antithrombotic, and antiproliferative properties. However, cardiovascular risk factors including hyperglycemia, insulin resistance, dyslipidemia, hypertension, hyperhomocysteinemia, smoking, and psychological stress, can impair NO production and reduce its bioavailability. These disruptions disturb endothelial‐dependent vasodilation, setting the stage for the development of atherosclerosis.

Hemodynamic alterations also contribute to endothelial dysfunction.^[^ ^3^ ^]^ Atherosclerotic plaques are not randomly distributed but instead tend to be seen in regions of disturbed laminar flow, including arterial bifurcations and curves. These hemodynamic disturbances can increase the retention of LDL in the arterial wall via matrix remodeling and affect the expression of shear stress response elements, both of which can compromise endothelial function.^[^ ^4^ ^]^ Recent research indicates that endothelial cells exhibit a variety of protective mechanisms when they are under stable laminar shear stress, including selectively upregulating cyclooxygenase‐2, manganese superoxide dismutase (SOD), and NO synthase, which helps protect against the development of atherosclerosis.^[^ ^5^ ^]^ Disturbed flow, however, triggers the persistent activation of proinflammatory pathways, upregulating leukocyte adhesion receptors [e.g., intercellular cell adhesion molecule‐1 and vascular cell adhesion molecule‐1 (VCAM‐1)] and proinflammatory cytokines and chemokines. These changes facilitate the recruitment and adhesion of immune cells and further promote atherosclerotic development.^[^ ^6^ ^]^

#### 2.1.2 Macrophage Heterogeneity and Functional Polarization

Macrophages play a key role in the initiation and progression of atherosclerosis (**Figure** **2**). During the early stages of the disease, circulating monocytes are recruited to areas of endothelial injury and adhere to endothelial cells under the influence of chemokines and adhesion molecules. These monocytes then transmigrate into the arterial intima, differentiating into macrophages in response to growth factors such as granulocyte‐macrophage colony‐stimulating factor and macrophage colony‐stimulating factor. These differentiated macrocytes exhibit substantial heterogeneity, as their phenotypes are largely determined by the local cytokine environment. For example, Th1‐derived interferon‐γ promotes the differentiation of classically activated (M1) macrophages, while Th2‐derived interleukin (IL)‐4 and IL‐13 promote the formation of alternatively activated (M2) macrophages.^[^ ^7^ ^]^

Macrophage phenotypes have distinct gene expression profiles and metabolic adaptations that shape their functional roles in atherosclerosis. After being activated by cholesterol, M1 macrophages secrete a range of proinflammatory cytokines [e.g., IL‐1, IL‐6, and tumor necrosis factor (TNF)‐α] as well as matrix metalloproteinases (MMPs). These factors amplify inflammatory responses, degrade extracellular matrix components, and lead to plaque vulnerability. Metabolically, M1 macrophages rely largely on aerobic glycolysis for rapid adenosine triphosphate (ATP) production. This metabolic shift upregulates the pentose phosphate pathway, which generates NADPH to support the production of reactive oxygen species (ROS) and NO via NADPH oxidase and inducible NO synthase, respectively. The tricarboxylic acid cycle is also disrupted at two key enzymatic steps—isocitrate dehydrogenase and succinate dehydrogenase (SDH)—in M1 macrophages, leading to the accumulation of citrate and succinate. Citrate is transported to the cytosol, where it is metabolized via the citrate‐pyruvate cycle to generate NADPH, which in turn supports the synthesis of ROS and NO. Concurrent enhancements in glycolysis lead to proton accumulation in the mitochondrial intermembrane space, resulting in an elevated mitochondrial membrane potential. The accumulated succinate can be oxidized by SDH, with reverse electron transport promoting the production of mitochondrial ROS (mROS). The combined effects of succinate accumulation and increased mROS stabilize hypoxia‐inducible factor‐1α (HIF‐1α), which drives IL‐1β expression.^[^ ^8^ ^]^

In contrast to M1 macrophages, M2 macrophages are characterized by their anti‐inflammatory and tissue repair functions. These cells secrete anti‐inflammatory cytokines [e.g., IL‐10 and transforming growth factor (TGF)‐β], which dampen inflammatory responses and promote plaque stabilization. Metabolically, M2 macrophages rely on oxidative phosphorylation and fatty acid oxidation, which provide a sustained, efficient energy source to support their reparative functions. M2 macrophages also exhibit elevated arginase expression, which both increases NO production and promotes the synthesis of collagen precursors, thereby facilitating tissue remodeling. These metabolic adaptations allow M2 macrophages to resolve arterial inflammation and stabilize plaques. The metabolic differences between M1 and M2 macrophages highlight their distinct roles in atherosclerosis. Targeted manipulation of these metabolic pathways could help regulate macrophage activity and modulate inflammatory responses in atherosclerosis.^[^ ^9^ ^]^

#### 2.1.3 Phenotypic Modulation and Transdifferentiation of VSMCs

VSMCs exhibit remarkable plasticity. They can adopt different phenotypes in response to their positions within atherosclerotic plaques. This phenotypic versatility is a crucial element of the formation, progression, and stability of atherosclerotic lesions.

In physiologic conditions, VSMCs maintain a “contractile phenotype” which helps preserve vascular tone. This phenotype is characterized by low‐frequency cellular proliferation and the sustained expression of contractile proteins such as α‐smooth muscle actin (α‐SMA), calponin, and smooth muscle myosin heavy chain. However, in the early stages of atherosclerosis, endothelial dysfunction triggers the release of platelet‐derived growth factor, which promotes the migration and proliferation of VSMCs from the vascular media to the intima. Under the influence of TGF‐β, some VSMCs undergo phenotypic modulation and transition into a “synthetic phenotype.” In this state, VSMCs secrete essential components of the extracellular matrix (ECM), including collagen, elastin, and proteoglycans, contributing to plaque growth and maturation. As atherosclerosis progresses, VSMCs take on a more complex role. VSMC‐synthesized ECM is essential for the formation and maintenance of the atherosclerotic fibrous cap, and protects against plaque rupture. However, VSMC apoptosis can undermine the structural integrity of the fibrous cap, leading to the accumulation of cellular debris, exacerbating inflammation, and increasing plaque vulnerability.^[^ ^10^ ^]^

In advanced atherosclerotic plaques, some VSMCs undergo a further phenotypic shift, downregulating traditional smooth muscle markers such as α‐SMA and upregulating macrophage markers like CD68. This transformation into macrophage‐like cells enables VSMCs to internalize lipids and form smooth‐muscle‐cell‐derived foam cells. Recent research suggests that up to 50% of foam cells in human atherosclerotic plaques may originate from VSMCs, not macrophages, as was traditionally believed.^[^ ^11^ ^]^ These macrophage‐like VSMCs can also secrete proinflammatory cytokines (such as IL‐6 and TNF‐α) and MMPs. These factors further exacerbate inflammation, degrade the ECM, and destabilize the plaque. Thus, by both contributing to foam cell formation and driving inflammation, VSMCs play a dual role in promoting plaque vulnerability.^[^ ^12^ ^]^

### 2.2 Key Pathological Processes Underlying Lesion Development

#### 2.2.1 Oxidative Stress: From Redox Imbalance to Cellular Injury and Plaque Destabilization

ROS are produced via various pathways, including NADPH oxidase (NOX), the mitochondrial electron transport chain, xanthine oxidase, and eNOS. Traditionally, ROS were viewed as byproducts of cellular metabolism, but modern research has highlighted their active role in regulating physiological processes. Among ROS, hydrogen peroxide (H_2_O_2_) is particularly notable because it can modulate signaling pathways via the reversible oxidation of specific protein targets and can thus influence cellular proliferation, differentiation, and migration.^[^ ^13^ ^]^ Low levels of ROS can facilitate the oxidation of specific cysteine residues in Kelch‐like ECH‐associated protein 1, a transcription factor that binds to antioxidant response elements to initiate the transcription of genes encoding antioxidant enzymes such as SOD, glutathione peroxidase (GPX), and catalase (CAT). These enzymes help mitigate oxidative damage and maintain cellular integrity. ROS can also activate AMP‐activated protein kinase (AMPK), a master regulator of cellular energy metabolism. AMPK activation indirectly inhibits the mammalian target of rapamycin (mTOR), promoting autophagy, which further aids in the removal of damaged organelles and proteins and helps maintain cellular homeostasis. This series of responses is referred to as “oxidative eustress.”^[^ ^14^ ^]^ However, excessive ROS production can overwhelm cellular antioxidant defenses, leading to oxidative stress.

Oxidative stress deeply impairs proper endothelial cell functioning. Tetrahydrobiopterin (BH_4_), a crucial eNOS cofactor, is highly sensitive to oxidative stress. Under oxidative conditions, reduced BH_4_ levels lead to the uncoupling of eNOS, resulting in the production of superoxide (O_2_^−^) instead of NO. Superoxide rapidly reacts with NO to form peroxynitrite (ONOO^−^), which further decreases NO's bioavailability and exacerbates oxidative stress.^[^ ^15^ ^]^ Diminished NO levels and the accumulation of ONOO^−^ both lead to endothelial dysfunction, which is characterized in this context by impaired endothelial‐dependent vasodilation, enhanced inflammation, and increased thrombus formation.^[^ ^16^ ^]^ A compromised endothelial barrier also facilitates LDL's entry into the arterial intima, where it is retained in the extracellular matrix through interaction with proteoglycan.^[^ ^17^ ^]^ Although native LDL is internalized and metabolized by LDL receptors, this classical pathway is insufficient for foam cell formation on its own. As intracellular cholesterol levels increase, negative feedback downregulates LDL receptor expression, limiting further LDL uptake. Even in individuals with familial hypercholesterolemia, who lack functional LDL receptors, atherosclerosis persists, indicating that alternative mechanisms must be involved.^[^ ^18^ ^]^ Oxidative stress induces the oxidation of unsaturated fatty acids and apolipoprotein B‐100 in LDL, generating oxidized low‐density lipoprotein (oxLDL). Unlike native LDL, oxLDL is internalized by macrophage scavenger receptors (e.g., SR‐A and CD36), which are not driven by intracellular cholesterol levels. This continuous oxLDL uptake leads to excessive cholesterol accumulation, resulting in foam cell formation.^[^ ^19^ ^]^

Oxidative stress impacts numerous intracellular macromolecules, including DNA, lipids, and proteins. It leads to cellular dysfunction, apoptosis, and pyroptosis. DNA damage activates the transcription factor p53, which upregulates proapoptotic genes such as *BAX* and *FAS*. These genes trigger apoptosis via the mitochondrial pathway and the death receptor pathway, leading to programmed cell death.^[^ ^20^ ^]^ Mitochondrial DNA (mtDNA) is particularly vulnerable to oxidative damage because of its proximity to the sites of ROS production within the mitochondria and its limited repair capacity. When mtDNA leaks into the cytoplasm, it is detected by cyclic GMP‐AMP synthase (cGAS), which produces cyclic GMP–AMP (cGAMP). cGAMP then activates inflammatory pathways such as stimulator of interferon genes, TANK‐binding kinase 1, interferon regulatory factor 3, NF‐κB, and nucleotide‐binding oligomerization domain‐like receptor protein 3 (NLRP3), leading to pyroptosis.^[^ ^21^ ^]^ ROS concurrently attacks polyunsaturated fatty acids in cellular membranes, generating lipid peroxides (LOOH). If LOOH are not completely cleared by GPX4, they will initiate a lipid peroxidation chain reaction in the presence of iron. This process generates alkoxyl radicals (R•) and peroxyl radicals (ROO•), which further attack membrane lipids. The accumulation of lipid peroxidation products severely damages cellular membrane integrity and can ultimately compromise cellular function and lead to cell death.^[^ ^22^ ^]^ Finally, oxidative stress also impairs endoplasmic reticulum (ER) protein folding capacity, leading to the accumulation of unfolded proteins and triggering the unfolded protein response. When this stress exceeds cellular regulatory capacity, apoptosis can be induced.^[^ ^23^ ^]^

Several studies have suggested that oxidative stress influences the development of atherosclerosis via specific signaling pathways. Work by Meng et al. indicated that PM2.5‐induced oxidative stress can disrupt intracellular Ca^2^⁺ homeostasis via the ryanodine receptor 2 pathway, which activates other apoptotic pathways and exacerbates myocardial injury in hyperlipidemic mice.^[^ ^24^ ^]^ Li et al.’s work suggested that inhibiting the ROS‐checkpoint kinase 2 axis can prevent the excessive inflammatory activation of macrophages and inhibit M2 to M1 macrophage polarization.^[^ ^25^ ^]^ Shen et al.’s findings suggested that the presence of increased oxygen free radicals in plaques can activate the cGAS/mTOR pathway via mitochondrial DNA damage, promoting M2 to M1 polarization and inhibiting macrophage autophagy.^[^ ^26^ ^]^

#### 2.2.2 Chronic Inflammation: Lipid‐Driven Immune Activation and Impaired Resolution

Inflammatory responses in atherosclerosis are complex and are driven by lipids.^[^ ^27^ ^]^ Lysophosphatidylcholine, which is released during the oxidative modification of LDL, has potent chemotactic properties for monocytes and T lymphocytes.^[^ ^28^ ^]^ This process is significantly amplified when oxLDL binds to the endothelial receptor lectin‐like oxLDL receptor 1 (LOX‐1), leading to a dose‐dependent increase in ROS generation.^[^ ^29^ ^]^ The ensuing ROS production activates the NF‐κB signaling pathway and the NLRP3 inflammasome, upregulating adhesion molecules, proinflammatory cytokines, and monocyte chemotactic protein‐1 (MCP‐1). Taken together, these processes all promote monocyte recruitment and adhesion.^[^ ^30^ ^]^

Monocytes that pass through the subendothelium differentiate into macrophages. These macrophages can internalize lipoproteins, especially oxLDL, through scavenger receptors such as SR‐A and CD36. Within the macrophages, cholesterol esters (CEs) are hydrolyzed into free cholesterol (FC) and fatty acids (FAs). FC is transported to the ER and other cellular compartments via Niemann–Pick‐C1 (NPC1) and NPC2‐mediated pathways.^[^ ^31^ ^]^ In the ER, FC is re‐esterified into CE by the enzyme acyl‐CoA acyltransferase, and then stored in lipid droplets, forming the characteristic “foam cells” seen in atherosclerotic lesions. In the early stages of atherosclerosis, accumulated CE can be hydrolyzed by neutral cholesterol ester hydrolase. Alternatively, through a process known as lipophagy, lipid droplets are degraded via autophagy, and the cholesteryl esters stored within them are hydrolyzed to FC by lysosomal acid lipase.^[^ ^32^ ^]^ FC is then transported to the plasma membrane, where it is effluxed to apolipoprotein A1 (apoA1) or high‐density lipoprotein (HDL) particles via ATP‐binding cassette transporter (ABCA1/ABCG1). In the most advanced stages of atherosclerosis, however, the FC reesterification processes can be compromised, leading to FC accumulation and the formation of cholesterol crystals within lysosomes. These crystals destabilize the lysosomal membrane, which releases cathepsin B and subsequently activates the NLRP3 inflammasome.^[^ ^33^ ^]^ Additionally, when oxLDL binds to CD36 receptors on endothelial cells and macrophages, it initiates a complex signaling cascade involving Toll‐like receptors (TLR4/TLR6), myeloid differentiation primary response protein 88, and Toll/interleukin‐1 receptor domain‐containing adaptor protein inducing interferon‐β, which culminates in the activation of NF‐κB. This promotes the transcription of pro‐IL‐1β and pro‐IL‐18.^[^ ^34^ ^]^ Further, the activated NLRP3 inflammasome recruits caspase‐1 via the caspase recruitment domain (ASC) adaptor protein, which cleaves pro‐IL‐1β and pro‐IL‐18 into their active forms, IL‐1β and IL‐18. Caspase‐1‐mediated cleavage of the Gasdermin D protein generates a N‐terminal fragment, which compromises cellular membrane integrity and triggers pyroptosis—a form of lytic, inflammatory cell death that amplifies local inflammatory responses and contributes to plaque destabilization.^[^ ^35^ ^]^

Acute inflammatory responses generally transition to a resolution phase after activation. This transition is impaired in advanced atherosclerosis cases, however, resulting in a chronic, persistent inflammatory state. Foam cells are prone to apoptosis because they are already subject to oxidative stress, ER stress,^[^ ^36^ ^]^ and death receptor signaling at baseline.^[^ ^37^ ^]^ In early atherosclerosis, apoptotic cells are typically cleared by neighboring M2 macrophages via efferocytosis. This process facilitates inflammatory resolution by releasing anti‐inflammatory cytokines and bioactive lipids. These proresolving mediators bind to specific receptors and inhibit the production of proinflammatory cytokines and MMPs, activate survival pathways to counteract ER‐stress‐induced apoptosis, and promote efferocytosis. They also stimulate fibroblasts to produce collagen, which is an essential protection against plaque destabilization.^[^ ^27a^ ^]^ In advanced atherosclerosis, however, foam cells express chemotactic molecules (e.g., netrin‐1, Unc‐5 netrin receptor B, semaphorin 3E, and cadherins)^[^ ^38^ ^]^ and secrete excessive proinflammatory cytokines, which disrupts the balance between M1 and M2 cells and exacerbates the proinflammatory state. Foam cells also secrete MMPs that degrade critical ECM proteins like collagen and elastin, weakening the fibrous cap and increasing plaque vulnerability. Compromised efferocytosis leads to the further accumulation of uncleared apoptotic cells, which eventually undergo secondary necrosis.^[^ ^39^ ^]^ This process, coupled with cellular death pathways such as ferroptosis and pyroptosis, releases damage‐associated molecular patterns (e.g., high mobility group box‐1 protein, ATP, DNA, and RNA), which are recognized by macrophage pattern recognition receptors. These interactions amplify inflammation and destabilize the plaques.

## 3 Therapeutic Limitations of Conventional Atherosclerosis Treatments

Improved understandings of cholesterol biosynthesis pathways and compelling epidemiological evidence which linked LDL levels to heightened cardiovascular risk firmly established 3‐hydroxy‐3‐methylglutaryl‐CoA reductase (HMG‐CoA reductase) inhibitors, also known as “statins,” as the cornerstone of lipid‐lowering therapies and the gold standard for secondary prevention in coronary artery disease. Statins effectively reduce circulating LDL‐C levels and lower the incidence of cardiovascular events.^[^ ^40^ ^]^ In addition to their lipid‐lowering effects, however, statins also exhibit potent anti‐inflammatory properties. They enhance endothelial function, inhibit macrophage and VSMC proliferation, suppress foam cell formation, and have strong antithrombotic activities. These multifaceted actions contribute to their central role in contemporary atherosclerosis treatment.

The long‐term, systemic use of high‐dose statins is also associated with adverse effects, however. Common side effects such as muscle pain and elevated liver enzymes can compromise patient adherence or lead to the discontinuation of treatment.^[^ ^41^ ^]^ The limited oral bioavailability and first‐pass metabolism also constrain their therapeutic effectiveness. Several large randomized controlled trials also demonstrated that, although statin‐based therapies—alone or in combination with cholesteryl ester transfer protein inhibitors, cholesterol absorption inhibitors, or proprotein convertase subtilisin/kexin type 9 (PCSK9) inhibitors—effectively lower LDL‐C levels, a substantial residual risk for cardiovascular events remains among patients with atherosclerotic vascular disease.^[^ ^42^ ^]^ Even when LDL‐C levels are reduced below 70 mg dL^−1^, ≈20% of patients continue to experience plaque progression.^[^ ^43^ ^]^ These observations underscore the inadequacy of current lipid‐lowering strategies to fully mitigate the atherosclerotic burden.

As the pathophysiological mechanisms underlying atherosclerosis continue to be characterized, inflammation has emerged as a central driver of its progression. Epidemiological studies consistently support a strong relationship between elevated inflammatory marker levels [e.g., C‐reactive protein (CRP) levels] and increased atherosclerotic cardiovascular disease risk.^[^ ^44^ ^]^ Landmark trials such as CANTOS demonstrated that IL‐1β with canakinumab significantly reduced the incidence of major adverse cardiovascular events (MACE) independent of lipid levels.^[^ ^45^ ^]^ Similarly, tocilizumab, an IL‐6 receptor antagonist, has shown endothelial benefits in inflammatory disease settings despite its prolipidemic effects.^[^ ^46^ ^]^ Colchicine, a well‐established anti‐inflammatory agent, has also shown clinical benefits in both acute and chronic coronary artery disease settings.^[^ ^47^ ^]^ These findings highlight the powerful potential of anti‐inflammatory treatments in cardiovascular disease.

Clinical translation of anti‐inflammatory therapies remains a challenge, however. For instance, low‐dose methotrexate treatment failed to reduce IL‐1β, IL‐6, or CRP levels or decrease MACEs in patients with stable atherosclerosis.^[^ ^48^ ^]^ Other agents, including p38‐mitogen‐activated protein kinase pathway inhibitors (e.g., losmapimod) and lipoprotein‐associated phospholipase A2 inhibitors (e.g., darapladib, varespladib), have also yielded disappointing results in patients with ischemic heart disease.^[^ ^49^ ^]^ Additionally, although cytokine‐targeting biologics can dampen inflammation, their immunosuppressive effects may heighten infection risk. These outcomes reflect the complexity of immune‐modulating therapies, as well as the need for precise, site‐specific treatments.

Given oxidative stress’ critical role in driving atherosclerosis, antioxidant therapies have also attracted considerable interest. Classic antioxidants such as vitamins C and E, polyphenols (e.g., resveratrol), probucol, and coenzyme Q have been extensively studied in these conditions.^[^ ^50^ ^]^ However, most clinical trials have failed to demonstrate meaningful cardiovascular benefits from antioxidant agents, largely due to their limited bioavailability and poor pharmacokinetic properties. The HPS Study, for example, reported no significant reduction in cardiovascular events with vitamin E or C supplementation.^[^ ^51^ ^]^

Collectively, these limitations—including residual cardiovascular risk despite intensive lipid‐lowering therapy, the nonspecific or suboptimal modulation of inflammation, and the limited efficacy of conventional antioxidant treatments, highlight a need for more targeted, effective, and safer pharmacological strategies in cardiovascular disease. In response to these challenges, NDDS has emerged as a promising platform that may be able to overcome the inherent shortcomings of traditional therapies (Figure 1).

The therapeutic efficiency of conventional drugs is often constrained by several factors: 1) they undergo nonspecific binding to plasma proteins (e.g., albumin), which reduces free drug serum concentrations; 2) they are rapidly cleared either hepatically or renally, which shortens therapeutic exposure; 3) they undergo immune‐mediated elimination via complement activation or adaptive immune recognition, leading to opsonization and mononuclear phagocyte system uptake; 4) they have suboptimal physicochemical properties (e.g., hydrophilicity, molecular weight >500 Da, or charges) which hinder transendothelial or cellular entry. NDDSs are designed to overcome these challenges by harnessing biocompatibility nanocarriers that shield therapeutic agents from enzymatic degradation and premature systemic clearance. Surface functionalization (e.g., ligand conjugation) and microenvironment‐responsive designs (e.g., pH‐/enzyme‐triggered release) enable site‐specific drug delivery and facilitate intracellular uptake. By enhancing drug accumulation at the site of interest and minimizing off‐target exposure, NDDSs improve therapeutic efficacy and reduce systemic toxicity. NDDS is also able to codeliver multiple drugs or bioactive molecules, which would enable concurrent modulation of several pathways that underlie atherosclerotic development. Some nanomaterials also have intrinsic therapeutic properties, such as anti‐inflammatory or antioxidant effects, which allow them to function as both carriers and active agents. These unique capabilities position NDDS as a promising platform for personalized and precision‐based therapy in atherosclerosis. An overview of current therapies and their limitations is presented in **Table** **1**.

**Table 1:** Current limitations and challenges in atherosclerosis treatment.

	Examples	Mechanism of action	Limitations
Lifestyle modifications	Healthy diet Weight control Physical activity Smoking cessation		Requires long‐term adherence Limited effectiveness in advanced stages Outcomes vary due to genetic and environmental factors
Lipid‐lowering therapy	Statins PCSK9 inhibitors Cholesterol absorption inhibitors	Inhibit cholesterol synthesis or absorption Enhance LDL‐C clearance Improve lipid profiles	“Statin intolerance” or “statin resistance” Residual risk for cardiovascular events Limited oral bioavailability
Anti‐inflammatory therapy	IL‐1β inhibitor (canakinumab) IL‐6 receptor inhibitor (tocilizumab) Colchicine	Reduce vascular inflammation Inhibit inflammatory signaling pathways	Efficacy unclear Potential toxicity Immunosuppressive risk
Antioxidant therapy	Vitamins C and E Polyphenols CoQ	Mitigate oxidative stress	Limited ability of single antioxidants to scavenge ROS Low bioavailability
Antiplatelet therapy	Aspirin P2Y12 receptor antagonists	Inhibit platelet aggregation Reduce thrombus formation	Increased bleeding risk
Surgical interventions	Percutaneous coronary intervention (PCI) Coronary artery bypass grafting (CABG)	Restore coronary blood flow	Procedural invasiveness Possibility of restenosis or stent thrombosis Limited surgical indications Cannot address systemic atherosclerosis

## 4 Strategies for Nanoparticle‐Based Drug Delivery in the Treatment of Atherosclerosis

### 4.1 Cell‐Targeted Nanoparticle for Atherosclerosis

#### 4.1.1 Macrophage Targeting Strategies

##### Inhibition of Monocyte Infiltration

Monocyte recruitment is a crucial early event in the development of atherosclerosis (Figure 2‐①). Persistent monocyte recruitment significantly increases the inflammatory burden of atherosclerotic plaques and counteracts natural inflammatory resolution. MCP‐1/C‐C motif chemokine ligand 2 (CCL2), a key chemokine, guides monocytes into atherosclerotic plaques in response to proinflammatory stimuli and tissue injury. Silencing the chemokine receptor C‐C motif chemokine receptor 2 (CCR2) reduces Ly‐6^high^ monocyte recruitment, which alleviates inflammation and left ventricular remodeling after myocardial infarctions.^[^ ^52^ ^]^ Building on these findings, Yin et al. developed nanoparticles encapsulating Bindarit, a potent MCP‐1/CCL‐2 inhibitor, using yeast‐derived capsules.^[^ ^53^ ^]^ These nanoparticles can be administered orally and subsequently target plaques using a “Trojan horse” strategy. After being absorbed by intestinal M cells, Ly6C^+^ monocytes transport them to peripheral blood. They are then distributed to atherosclerotic plaques, reducing monocyte recruitment and preventing plaque formation.

CD4^+^ T cells play a key role in activating monocytes and macrophages via the CD40–CD40L signaling pathway, which is a well‐established driver of atherosclerosis.^[^ ^54^ ^]^ Activated monocyte/macrophage CD40 recruits TNF receptor‐associated factor 6 (TRAF6) intracellularly, initiating a proinflammatory signaling cascade.^[^ ^55^ ^]^ Seijkens and co‐workers recently incorporated a TRAF6 inhibitor into recombinant HDL nanoparticles.^[^ ^56^ ^]^ Their innovative approach targeted the interaction between CD40 and TRAF6, which reduced CD40 and integrin expression on monocytes and thus inhibited monocyte recruitment. After six weeks of treatment, rHDL‐6877002 therapy was shown to reduce early atherosclerosis progression in ApoE^−/−^ mice.

##### Macrophage Clearance in Early Stage Atherosclerosis

Given the critical role of macrophages in atherosclerotic progression, there has been a growing interest in using nanoparticles to induce macrophage apoptosis and reduce macrophage burdens within early stage atherosclerotic plaques. Three innovative therapeutic modalities, photothermal, photodynamic, and chemodynamic therapies, have been repurposed from oncology to address this challenge and may offer new avenues for atherosclerosis treatment.

Photothermal therapy (PTT) leverages the light‐absorbing properties of certain agents to induce thermal necrosis in macrophages. The recent NANOM‐FIM trial demonstrated the potential of silica–gold nanoparticles to reduce atherosclerotic plaque size with a favorable safety profile.^[^ ^57^ ^]^ However, the nonspecific distribution of thermal energy remains a concern, as this may lead to unintended damage to surrounding tissues and structures.

By contrast, photodynamic therapy, also referred to as “photovascularization,” involves the administration of photosensitizers followed by activation with specific light wavelengths at the lesion site using a special catheter‐guided laser. When activated, the photosensitizer enters an excited state, transferring energy to surrounding molecular oxygen and generating ROS, which in turn induces cytotoxic effects. McCarthy et al. used dextran‐coated iron oxide magnetic nanoparticles (CLIO‐THPC) to deliver the photosensitizer tetrahydroxyphenylchlorin (THPC) to macrophages and achieved significant levels of macrophage apoptosis and plaque stabilization in murine models.^[^ ^58^ ^]^ This approach enhances therapeutic efficacy while minimizing skin toxicity, improving its translational potential.

Cao and co‐workers developed copper sulfide/titanium oxide heterostructure nanosheets modified with hyaluronic acid (HA) and polyethylene glycol (PEG) (HA‐HNSs).^[^ ^59^ ^]^ These nanosheets operate on the principles of sonodynamic therapy (SDT), where ultrasound locally activates the inorganic sonosensitizer titanium dioxide (TiO_2_) and generates ROS that induce macrophage apoptosis. Additionally, the downregulation of heat shock protein 90 reduces cellular tolerance of heat stress. Copper sulfide (CuS) serves as a photothermal agent, providing mild PTT in the near‐infrared II (NIR‐II) region. The synergistic effects of low‐intensity SDT and mild PTT then effectively induce apoptosis in macrophages.

Mu et al. followed the principles of chemodynamic therapy to design nanocatalyst by conjugating dopamine‐modified HA with iron ions and tannic acid complexes (HFTNPs).^[^ ^60^ ^]^ HFTNPs accumulate in inflammatory macrophages via the CD44 receptor. In cases of elevated intracellular ROS levels, ferrous ions catalyze the decomposition of hydrogen peroxide to produce hydroxyl radicals, leading to the apoptosis of inflammatory macrophages. Oxidized ferric ions are then reduced by tannic acid complexes, completing the cyclic process.

Although these strategies have been shown to be effective in improving early stage atherosclerosis and reducing plaque burden, inducing macrophage apoptosis in advanced atherosclerotic plaques may pose further challenges. Specifically, depleting the functional macrophage pool could actually exacerbate inflammation in later‐stage disease.

##### Regulation of Macrophage Polarization

The delicate balance between M1 and M2 macrophage polarization is a crucial determinant of atherosclerotic plaque outcomes. While M1 macrophages exacerbate inflammation and increase plaque vulnerability, M2 macrophages contribute to plaque stability by promoting inflammatory resolution and fibrosis. Thus, modulating the M1/M2 balance may help alleviate inflammation and slow atherosclerotic plaque progression (Figure 2‐②).

Macrophage phenotypes are regulated by a complex network of molecular signals, with the activation of transcription factors leading to heterogeneity in gene expression and thus functional diversity among macrophages.^[^ ^61^ ^]^ The nuclear response peroxisome proliferator‐activated receptor gamma (PPAR‐γ) has been recognized for its role in promoting monocyte differentiation into the M2 phenotype, which lowers the M1/M2 ratio and decreases inflammation within atherosclerotic plaques.^[^ ^62^ ^]^ This realization has led to the development of PPAR‐γ agonists, such as pioglitazone, which offer antiatherosclerotic benefits in addition to their beneficial effects on glucose and lipid metabolism. Wu et al. developed liposomes modified with phosphatidylserine (PtdSer) and DSPE–PEG2000–cRGDfK to deliver pioglitazone directly to atherosclerotic plaques (AP‐Lipo).^[^ ^63^ ^]^ AP‐Lipo targets plaques via interactions between the cRGDfK peptide and αvβ3 integrin receptors and then signals macrophages with “eat me” signals via surface PtdSer. This strategy facilitates the targeted delivery of pioglitazone to M1 macrophages, promoting the survival of anti‐inflammatory macrophages and slowing atherosclerotic progression.

Curcumin, a natural polyphenol with potent antioxidant, anti‐inflammatory, and lipid‐lowering properties, has been identified as a modulator capable of promoting M1 to M2. Fontana et al. encapsulated curcumin in lignin nanoparticles and coated the particles with tannic acid (TA)‐Fe(III) complexes and platelet‐membrane‐derived fragments (Curc@Lignin@TA@PL).^[^ ^64^ ^]^ The platelet membrane coating reduced immune activation and enhanced the biocompatibility and circulation time of the nanoparticles. Additionally, the coating facilitated targeted interactions with damaged blood vessels, promoting nanoparticle accumulation at the site of atherosclerotic plaques. In vitro studies showed that Curc@Lignin@TA@PL significantly downregulated NF‐ κb, TGF‐β1, IL‐6, and IL‐1 β in lipopolysaccharide (LPS)‐induced inflammatory endothelial cells. Similarly, Shi et al. used iron oxide nanoparticles as carriers, coupling them with PtdSer and 9‐carboxynonanoate (9‐CCN) to deliver “eat‐me” signals specifically to macrophages. This approach enabled the precise delivery of curcumin to macrophage cytoplasm and promoted polarization toward the M2 phenotype.^[^ ^65^ ^]^

Efforts have also been made to directly improve the concentration of proresolution mediators. Ac2‐26, a peptide mimetic of annexin A1, acts on the G‐protein‐coupled receptor formyl peptide receptor 2 to promote the polarization of M2‐like macrophages and facilitate inflammatory resolution. Encapsulating Ac2‐26 in collagen IV‐targeted polymer nanoparticles was shown to stabilize atherosclerotic plaques by increasing fibrous cap thickness and reducing oxidative stress and necrosis.^[^ ^66^ ^]^

Extensive prior research has highlighted miRNA's crucial role in atherosclerosis development, positioning it as a promising therapeutic target. The delivery of exogenous miRNA can promote the expression of beneficial miRNAs, while miRNA inhibitors can help suppress disease‐promoting miRNAs. However, challenges related to the stability, bioavailability, and biodistribution of these molecules continue to hinder their clinical applicability. NDDS may be able to address these challenges and improve the therapeutic potential of gene‐based therapies. Gao et al. loaded IL‐10 mRNA into self‐assembled nanoparticles composed of cationic lipid‐like molecules (G0‐C14) and a poly(lactide‐*co*‐glycolide) (PLGA)–PEG diblock copolymer (IL‐10 mRNA@M‐HNPs).^[^ ^67^ ^]^ Mannose modification was used to target M2 macrophages, which express high levels of mannose receptors when they are inside atherosclerotic plaques. Once internalized, these nanoparticles release therapeutic mRNA, which promotes IL‐10 expression. IL‐10 inhibits the production of proinflammatory cytokines, promotes M2 macrophage polarization, and reduces apoptosis and necrosis in atherosclerotic lesions, thus overall enhancing plaque stability.

**Figure 3:**
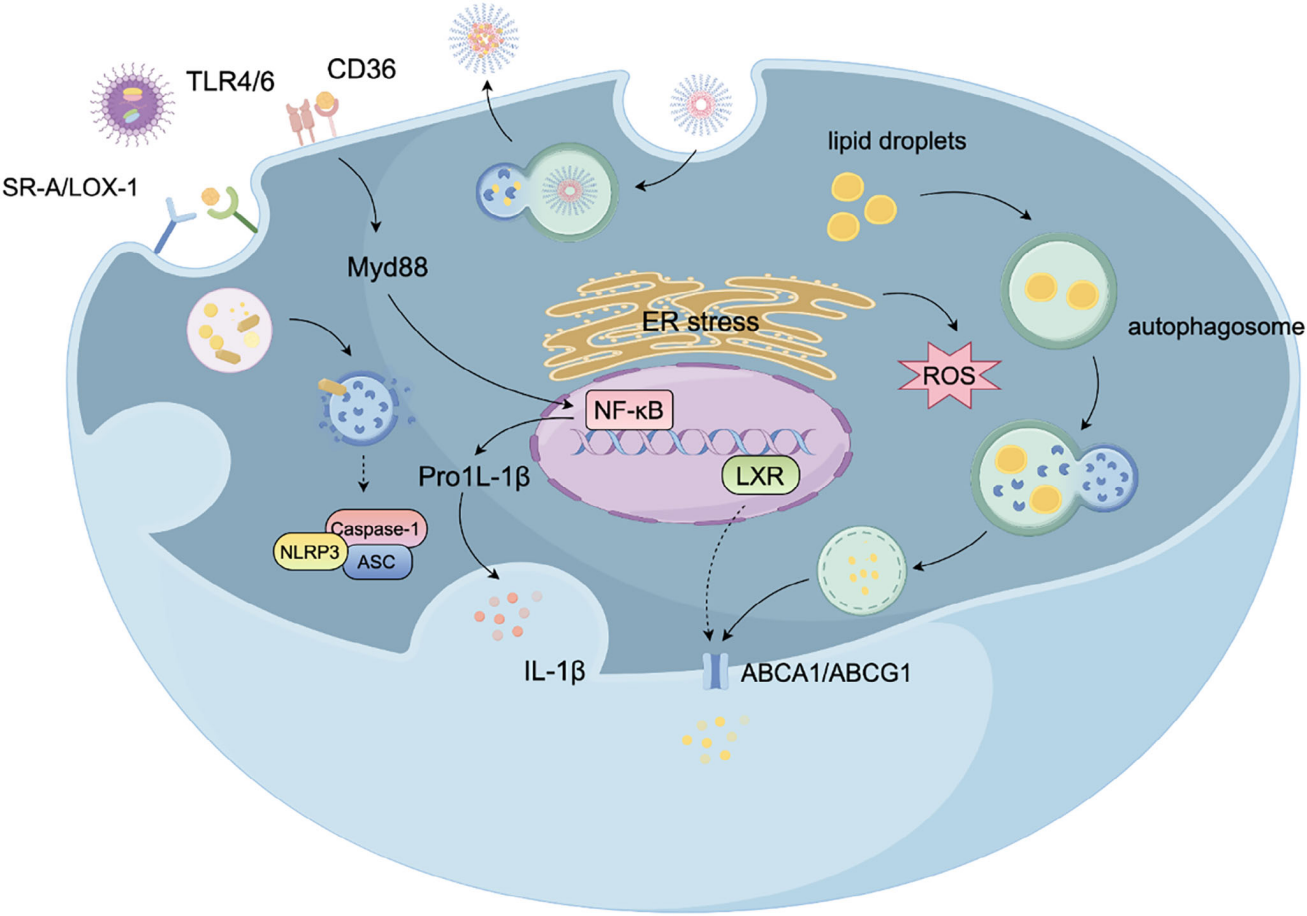
Nanoparticle mechanisms for regulating macrophage lipid metabolism. In atherosclerotic plaques, lipoproteins are taken up by macrophages via scavenger receptors and low‐density lipoprotein (LDL) receptor‐1 and broken down into fatty acids and free cholesterol (FC) in late endosomes. FC is re‐esterified in the endoplasmic reticulum (ER) and stored in lipid droplets, which can later be mobilized via neutral cholesterol ester hydrolase or lipophagy and exported via ATP‐binding cassette transporters. FC accumulation has two effects: it inhibits endogenous cholesterol synthesis and LDL receptor production while inducing oxysterol formation, which causes Liver X receptor (LXR) to activate ABCA1 for cholesterol efflux. Taken together, these processes help maintain homeostasis. However, excessive FC in late endosomes can form crystals, destabilizing lysosomal membranes and activating the NLRP3 inflammasome via cathepsin B release. FC deposition in the ER also disrupts its function, inducing ER stress, ROS release, and macrophage apoptosis. NDDSs regulate lipid metabolism in macrophages via two mechanisms. First, they competitively bind to macrophage surface scavenger receptors, such as scavenger receptor (SR)‐A1 and CD36, inhibiting oxidized low‐density lipoprotein (oxLDL) uptake and reducing intracellular cholesterol accumulation. Second, NDDSs enhance cholesterol efflux through several coordinated pathways: 1) activation of the LXR signaling pathway, which upregulates ATP‐binding cassette transporters ABCA1 and ABCG1, promoting RCT; 2) targeting of the mTOR signaling pathway, which induces lipophagy, facilitating the mobilization and degradation of intracellular lipid droplets; and 3) increasing solubility of cholesterol, which enables its direct removal from cells. Collectively, these mechanisms reduce foam cell formation and attenuate local inflammation, ultimately enhancing plaque stability and potentially facilitating regression.

###### 4.1.1.1 Regulation of lipid metabolism and autophagy in macrophages

Excessive lipid accumulation disrupts normal macrophage function and contributes to atherosclerotic progression. NDDS can specifically target and modulate lipid metabolism in macrophages by reducing lipoprotein uptake and promoting cholesterol efflux, thus alleviating lipid‐induced cytotoxicity. (**Figure** **3**) For example, Lewis et al. developed glycosylated amphiphilic molecules that mimic oxidized lipoproteins, enabling nanoparticles to competitively bind to macrophage scavenger receptors and reduce oxLDL uptake, which inhibits foam cell formation.^[^ ^68^ ^]^ LOX‐1 is another important oxLDL receptor. It has been associated with several pathological processes present in atherosclerosis, including cell formation, endothelial dysfunction, VSMC proliferation, collagen degradation, and platelet activation.^[^ ^30b^ ^]^ Zhao et al. designed a core–shell nanoparticle platform loaded with both atorvastatin and LOX‐1 siRNA.^[^ ^69^ ^]^ This platform uses HA and Apo‐I layers to selectively target endothelial cells and macrophages, respectively. By knocking down LOX‐1 expression in both cell types, this approach limits intracellular cholesterol accumulation, slows atherosclerotic progression, and may even reverse plaque formation.

**Figure 4:**
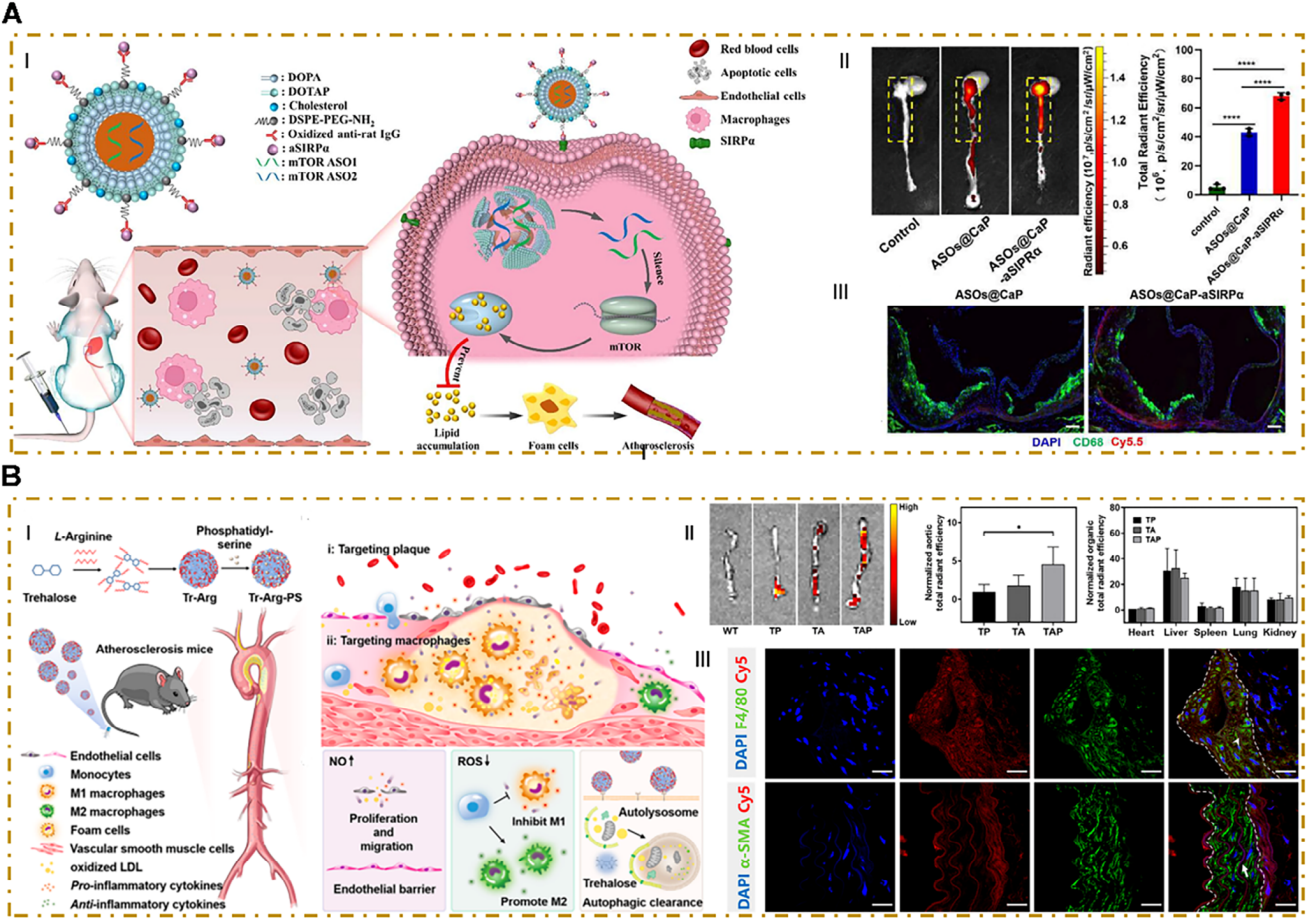
Macrophage‐targeted nanoparticles in atherosclerosis. A) I) Schematic illustration of macrophage‐mediated antiatherosclerosis therapy, which used aSIRPα‐modified, antisense‐oligonucleotide (ASOs)‐loaded CaP nanoparticles (ASOs@CaP–aSIRPα). II) Ex vivo fluorescence images and quantitative analysis of Cy5.5 fluorescent signal in the aorta showing the in vivo targeting capability of Cy5.5‐labeled ASOs@CaP–aSIPRα NPs in mice following intravenous injections. III) Immunofluorescence analysis of the colocalization of ASOs@CaP NPs or ASOs@CaP–aSIPRα NPs with CD68^+^ macrophages in aortic root cryosections. ASOs release curves from ASOs@CaP–aSIRPα NPs at pH 7.4, 6.5, and 5.0 over time. Reproduced with permission.^[^ ^71^ ^]^ Copyright 2025, ELSEVIER. B) I) Schematic illustration of the synthetic process of Tr–Arg–phosphatidylserine (PS) (TAP) nanomotors and the two‐stage‐targeted strategy for the comprehensive treatment of atherosclerosis. II) Ex vivo images and quantification showing accumulation of Cy5‐labeled TP NPs, TA, and TAP nanomotors in the aortas. III) Immunofluorescence images of the aortic arch showing the colocalization of TAP nanomotors with F4/80^+^ macrophages and α‐SMA^+^ VSMCs. Reproduced with permission.^[^ ^72^ ^]^ Copyright 2025,American Chemical Society.

A widely employed approach to combat atherosclerosis involves enhancing cholesterol efflux from macrophages. In advanced lesions, chronic lipid overload macrophage mTOR upregulation can suppress the process of autophagy,^[^ ^70^ ^]^ thus impairing autophagy‐mediated cholesterol clearance (lipophagy) and fostering macrophage apoptosis, which destabilizes plaques. mTOR can also drive macrophages toward the M1 phenotype by regulating glycolytic pathways and the NF‐κB signaling pathway. Rapamycin, a mTOR inhibitor, has a broad spectrum of pharmacologic effects. It has anti‐inflammatory, antimigratory, and antiproliferative properties and the capacity to activate autophagy. However, systemic rapamycin administration is associated with significant adverse effects, including immunosuppression, hypercholesterolemia, and hyperglycemia. Additionally, rapamycin's inhibitory action is limited to only one of the two intracellular mTOR proteins, which reduces its therapeutic efficacy. To address these limitations, Liu et al. developed a targeted nano‐bioconjugate that incorporated antisense oligonucleotides (ASOs) specific to mTOR.^[^ ^71^ ^]^ The resulting ASOs@CaP were surface‐modified with anti‐SIRPα antibodies (aSIRPα), enabling binding to the macrophage surface protein SIRPα. This modification facilitated the selective accumulation of ASOs@CaP in atherosclerotic plaques and simultaneously disrupted the CD47‐SIRPα axis to enhance the phagocytosis of apoptotic foam cells. The ASOs, after being integrated into the RNA‐induced silencing complex, downregulated mTOR expression, which boosted macrophage autophagy and reduced intracellular lipid accumulation (**Figure** **4**A). Similarly, Wu et al. developed a novel NO‐driven nanomotor, synthesized via a reaction of trehalose, l‐arginine, and phosphatidylserine.^[^ ^72^ ^]^ In the atherosclerotic microenvironment, l‐arginine reacts with NO synthase and ROS to generate NO, which propels the nanomotor toward the lesion via chemotaxis. The phosphatidylserine component then internalizes the nanomotor. As illustrated in Figure 4B, under this two‐step targeting strategy, Tr–Arg–phosphatidylserine (PS) (TAP) nanomotors efficiently accumulated in the aorta and colocalized with F4/80^+^ macrophages and α‐SMA^+^ VSMCs. Trehalose was shown to activate macrophage autophagy via the transcription factor EB pathway (and independent of the mTOR signaling pathway).^[^ ^73^ ^]^

Intracellular cholesterol is metabolized via enzymatic oxidation to yield oxysterols, which act as natural regulators of HMG‐CoA reductase and create a negative feedback loop to inhibit cholesterol synthesis.^[^ ^74^ ^]^ Oxysterols also function as endogenous ligands for liver X receptor (LXR). LXR activation upregulates the expression of ABCA1 and ABCG1, both of which enhance cholesterol efflux from macrophages and thus diminish foam cell formation.^[^ ^75^ ^]^ LXR activation also regulates inflammatory responses and induces arginase, which helps mitigate atherosclerosis progression.^[^ ^76^ ^]^ LXR has therefore, gained significant attention as a promising target for atherosclerosis treatment. Despite its therapeutic potential, the systemic administration of LXR agonists is associated with several adverse effects, including hypertriglyceridemia. Zhang et al. developed nanoparticles composed of self‐assembled poly(lactide‐*co*‐glycolide)‐*b*‐poly (ethylene glycol) (PLGA‐*b*‐PEG) to deliver the LXR agonist GW3965. This nanoparticle formulation showed enhanced efficacy in vitro and in vivo, which substantially reduced off‐target effects on lipid metabolism and hepatic function compared to the free drug.^[^ ^77^ ^]^

**Figure 5:**
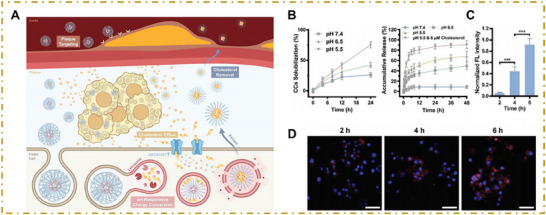
Dual‐track cholesterol clearance via pH‐responsive LXR agonist‐loaded nanoplatforms. A) Illustration of the dual‐track reverse cholesterol transport created by LPLCH, with atherosclerosis targeting and pH‐triggered lysosome escape. B) The cholesterol crystal (CC) carrying ability and in vitro release behavior of nanoparticle‐enabled LXR agonists after being incubated in liquids with various pHs. C,D) Fluorescent images and quantification of foam cells cocultured with (Rho) LPLCH for various amounts of time. Reproduced with permission.^[^ ^78^ ^]^ Copyright 2025, John Wiley and Sons.

Innovative nanoplatforms have emerged as powerful tools to treat atherosclerosis, as they enable a dual‐track approach that combines LXR agonist‐mediated reverse cholesterol transport (RCT) enhancement with the dissolution of cholesterol crystals. Chen et al. synthesized poly‐ε‐lysine covalently attached to cholesteryl moieties (PLC) to create self‐assembled micelles, which were then loaded with the LXR agonist T0901317. These micelles were coated with oxidized hyaluronic acid (oxHA) via pH‐sensitive imine bonds between poly‐ε‐lysine and oxHA, resulting in a targeting‐responsive nanoplatform, LPLCH.^[^ ^78^ ^]^ After interacting with CD44, LPLCH is internalized into macrophages in a time‐sensitive manner. The low pH in lysosomes protonates and breaks the imine bonds, causing the oxidized HA coat to be shed and triggering the “proton sponge effect,” which disrupts the lysosomal membrane.^[^ ^79^ ^]^ T0901317 is released simultaneously, is exchanged with cholesterol crystals, and is then carried away by the nanoparticles (**Figure** **5**).

Reconstituted high‐density lipoprotein (rHDL) nanoparticles mimic the structure of natural HDL, with apoA1 on their outer layers. These nanoparticles facilitate RCT by removing excess cholesterol from peripheral tissues and macrophages, which reduces the atherosclerotic plaque burden. Although initial studies on this technique were promising, a large randomized controlled trial in human participants suggested that a short‐term infusion of rHDL may not have satisfactory antiatherosclerotic effects.^[^ ^80^ ^]^ However, rHDL nanoparticles could still potentially serve as versatile drug carriers. For example, rHDL nanoparticles loaded with statins have demonstrated effective accumulation in atherosclerotic lesions and exhibited anti‐inflammatory effects in both long‐term (three months) low‐dose and short‐term (one week) high‐dose regimens without causing muscular or hepatic toxicity.^[^ ^81^ ^]^

Some nanomaterials can “pack up” lipids from cells “to go.” β‐cyclodextrin (β‐CD) is a unique nanomaterial with a hydrophobic cavity that allows for stable interactions with cholesterol. This property improves cholesterol stability and reduces cholesterol‐mediated cellular damage and inflammatory responses. Kim et al. introduced cargo‐switching nanoparticles with a core consisting of a β‐cyclodextrin–simvastatin inclusion complex.^[^ ^82^ ^]^ These nanoparticles exploit β‐CD's relatively higher affinity for cholesterol compared to statins and enable “cargo switching,” where cholesterol displaces simvastatin. This switch enables cholesterol clearance and releases the statin to further affect therapeutic results. Ma et al. developed a more comprehensive lipid management platform by constructing a complex composed of T0901317, prednisolone, and β‐CD. This complex, as well as a polymer photoacoustic probe, was encapsulated in a ROS‐responsive polymer (poly(2‐methylthio ethanol methacrylate), PMEMA) and coated with oxidized HA modified with a MMP‐9‐sensitive peptide.^[^ ^83^ ^]^ This nanoplatform, PLCDP@PMH, targets atherosclerotic CD44 receptors. When the nanoplatform encounters elevated ROS and/or MMP‐9 levels, it releases its payload, including the photoacoustic probe and the LCDP complex. The photoacoustic probe enhances imaging of atherosclerotic plaques, and the LCDP complex suppresses M1 macrophage polarization, enhances ABCA1/G1‐mediated lipid efflux, and promotes lipolysis, thus offering a comprehensive approach to lipid management.

Methotrexate (MTX), which is traditionally used in oncology and rheumatoid arthritis, has protective effects in atherosclerosis. MTX induces adenosine release, which upregulates the expression of cholesterol hydroxylase and ABCA1 and thus promotes cholesterol efflux.^[^ ^84^ ^]^ Zhu et al. developed a biomimetic nanocarrier by loading MTX onto dopamine‐modified β‐CD and covering it with macrophage membranes, resulting in MM@DA–pCD@MTX.^[^ ^85^ ^]^ This biomimetic nanocarrier uses a macrophage membrane coating to “home” the system to inflammatory endothelial cells within atherosclerotic lesions. Upon arrival, MTX is released and, along with β‐CD, synergistically enhances RCT, which inhibits foam cell formation. Additionally, dopamine, which is incorporated into the nanocarrier, retains its antioxidant properties, effectively scavenging ROS within the plaque and alleviating oxidative stress.

For gene therapy, Nguyen et al. used a chitosan polymer nanoparticle platform to deliver miR‐206 and miR‐223, two miRNAs that are known to enhance ABCA1 expression.^[^ ^86^ ^]^ In a parallel approach, Li et al. developed pH‐responsive cyclodextrin‐derived nanoparticles (RAAM NPs) which were specifically designed to deliver antisense oligonucleotides targeting miR‐33.^[^ ^87^ ^]^ miR‐33 is a well‐known regulator of cholesterol metabolism. It suppresses the expression of ABCA1 and ABCG1 in macrophages and thus inhibits cholesterol efflux to apolipoprotein A1 (Apo A‐I). By silencing miR‐33, the RAAM NP platform both facilitated RCT and modulated vascular adaptive immune responses. Cui et al. discovered that Epsin promotes CD36‐mediated lipid uptake and facilitates ABCG1 degradation, thus inhibiting ABCG1‐mediated cholesterol efflux. They also developed S2P nanoparticles for the targeted delivery of Epsin1/2 siRNA (S2PNP‐siEpsin1/2), showing that silencing Epsin in macrophages could alleviate inflammation, reduce necrotic core areas, and accelerate atherosclerotic regression.^[^ ^88^ ^]^

###### Revitalizing Efferocytosis

**Figure 6:**
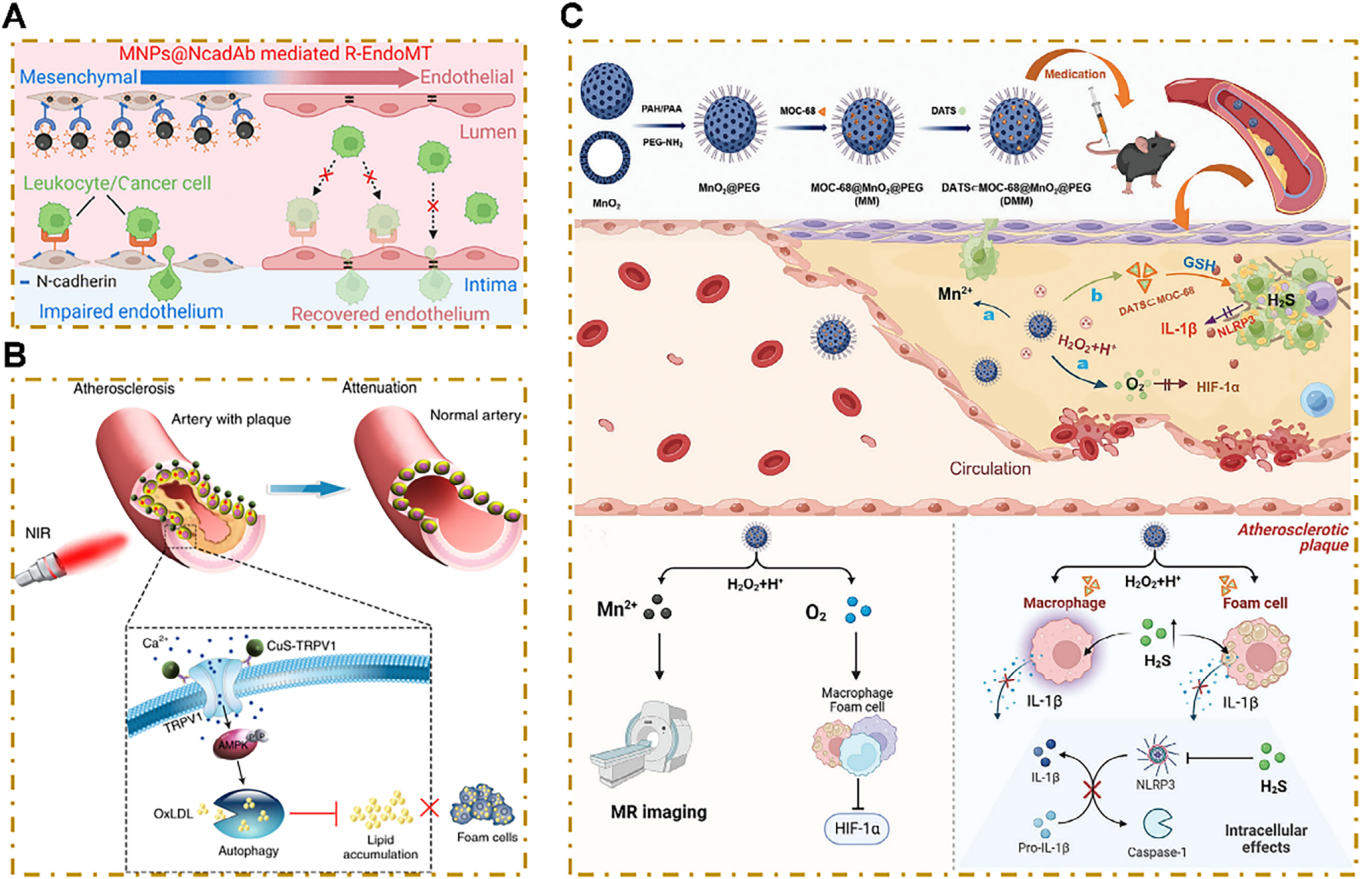
Nanoparticles targeting endothelial cells, smooth muscle cells, or inflammation. A) Schematic illustration of “Reversed EndoMT” (R‐EndoMT) in endothelial cells, which is mediated by MNPs@NcadAb. Reproduced with permission.^[^ ^99^ ^]^ Copyright 2025, American Chemical Society. B) Illustration of CuS–TRPV1 switch for the photothermal activation of TRPV1 signaling to attenuate atherosclerosis. Reproduced with permission.^[^ ^102^ ^]^ Copyright 2025, Springer Nature. C) Schematic illustration of the synthesis and delivery of DATS‐loaded MOC‐68‐based MnO_2_@PEG nanoparticle for atherosclerosis therapy. Reproduced with permission.^[^ ^110^ ^]^ Copyright 2025, John Wiley and Sons.

As cells approach apoptosis, they release “find‐me” signals, including lysophosphatidylcholine, to attract phagocytes and downregulate “do not eat me” signals, such as CD47, which inhibit phagocytosis. Proresolving macrophages (M2 macrophages) recognize and bind to “eat me” signals, such as PS, on the surface of apoptotic cells, initiating efferocytosis (Figure **2‐⑥**).^[^ ^89^ ^]^ Recent studies have shown that sustained upregulation of CD47 in human atherosclerotic plaques, particularly in the necrotic cores, is a key factor that disrupts efferocytosis. Blocking CD47 with antibodies can restore the clearance of diseased vascular tissues both in vitro and in atherosclerotic mouse models, reducing necrotic core sizes.^[^ ^90^ ^]^ However, systemic CD47 blockade can also have unintended consequences, such as increased splenic phagocytosis of red blood cells. This can lead to anemia and potentially exacerbate ischemia in patients with atherosclerotic disease. To address these challenges, Flores et al. developed a targeted delivery system using polyethylene‐glycol‐functionalized single‐walled carbon nanotubes to deliver Src homology 2 domain‐containing phosphatase‐1 inhibitors (SHP‐1i).^[^ ^91^ ^]^ SHP‐1 is a downstream effector molecule of the CD47 receptor signal‐regulatory protein‐α (SIRPα). These nanotubes act as “Trojan horses,” targeting inflammatory monocytes (Ly‐6C^hi^) and enhancing efferocytosis via regulation of the CD47–SIRPα–SHP1 axis within atherosclerotic plaques.

Another promising strategy targets calcium/calmodulin‐dependent protein kinase γ (CaMKIIγ) in macrophages. CaMKII activation has been seen in symptomatic atherosclerotic plaques and other advanced lesions in mice. Mice with myeloid‐specific CaMKII deficiency have smaller necrotic cores and thicker fibrous caps, indicating improved plaque stability and efferocytosis. Mechanistic studies have shown that CaMKIIγ deficiency increases ATF6 expression. ATF6 is a transcription factor that induces LXRα and upregulates the MerTK receptor, which is closely related to efferocytosis.^[^ ^92^ ^]^ To translate these findings into a therapy, PLGA and lipid–PEG nanoparticles, modified with S2P, were engineered to deliver siRNA targeting the *Camk2g* gene, which encodes CaMKIIγ. This strategy has shown promising results. It enhanced efferocytosis, reduced necrotic core areas, and increased fibrous cap thickness in atherosclerotic mouse models, thus promoting overall plaque stability.^[^ ^93^ ^]^ It enhanced efferocytosis, reduced necrotic core areas, and increased fibrous cap thickness in atherosclerotic mouse models, thus promoting overall plaque stability.

Chuang et al. developed hybrid switch receptor macrophages equipped with ROS‐responsive HPβ‐CD lipid nanoparticles (β‐CD LNPs).^[^ ^94^ ^]^ In this design, the anti‐CD47 chimeric antigen receptor (CAR) acts as a hybrid switch receptor that recognizes CD47 overexpression on apoptotic cells. It bypasses the inhibitory SIRPα–CD47 signaling pathway by transmitting proengulfment activation signals and enhances efferocytosis. Additionally, in the highly oxidative environment of atherosclerotic plaques, HPβ‐CD released from β‐CD LNPs dissolves cholesterol crystals and shifts cholesterol metabolism toward oxysterol production, thus enhancing LXR activation. This metabolic reprogramming supports apoptotic cell clearance, downregulates proinflammatory cytokine expression, and facilitates cholesterol efflux, all of which contribute to plaque stabilization. The dual action of efferocytosis promotion and cholesterol metabolism enhancement positions CAR macrophages as sophisticated therapeutic tools for atherosclerotic management.

#### 4.1.2 Regulation of Endothelial Function and Phenotypic Transformation of VSMC

Restoring endothelial function would be a critical therapeutic achievement in atherosclerosis management. NO is a major mediator of endothelial function, but its bioavailability is often compromised in atherosclerosis because of endothelial dysfunction. Wang et al. developed a targeted delivery system that used polysialic acid (PSA) to deliver budesonide and l‐arginine (BUD–l‐Arg@PSA) to activated endothelial cells.^[^ ^95^ ^]^ PSA binds to E‐selectin, which is overexpressed on inflamed endothelial cells, enabling precise targeting. l‐arginine serves as a substrate for eNOS, which converts it into NO, thereby enhancing endothelial function. Additionally, budesonide, a glucocorticoid, suppresses the NF‐κB pathway and induces eNOS expression, further promoting NO production. This dual‐action approach restores endothelial functioning and reduces inflammation.

RAGE is a transmembrane receptor that plays a pivotal role in amplifying inflammation in atherosclerosis. Its activation triggers the production of several proinflammatory cytokines, chemokines, and cell adhesion molecules, all of which increase leukocyte recruitment and promote atherosclerotic plaque progression. RAGE is also recognized by the leukocyte integrin Mac‐1, which further facilitates the adhesion and infiltration of inflammatory cells into the vascular wall. Mocanu et al. developed cationic PEGylated liposomes targeted to P‐selectin (Psel‐lipo/shRAGE lipoplexes) to deliver short hairpin RNA (shRNA), which specifically targeted RAGE.^[^ ^96^ ^]^ In ApoE^−/−^ mice, Psel‐lipo/shRAGE lipoplexes reduced RAGE‐signaling‐related inflammatory processes, delayed atherosclerotic progression, and did not have any significant adverse effects.

Addressing endothelial‐to‐mesenchymal transition (EndoMT) is another innovative therapeutic target. EndoMT, which involves the transformation of endothelial cells into mesenchymal‐like cells, is a major process in cardiovascular health and disease. In the context of atherosclerosis, inflammation, oxidative stress, and altered shear stress can activate the TGF‐β signaling pathway within endothelial cells and trigger inappropriate EndoMT.^[^ ^97^ ^]^ This transformation compromises normal endothelial function, as these cells morph into fibrogenic mesenchymal cells, which increase extracellular matrix production and augment migratory capacity. These alterations result in intimal thickening and endothelial barrier disintegration. Studies have also shown that EndoMT‐derived cells can upset the collagen–MMP balance within plaques, fostering a transition toward a more unstable phenotype that is prone to rupture.^[^ ^98^ ^]^ To counteract this process, Liu et al. engineered N‐cadherin‐targeting melanin nanoparticles. These nanoparticles were isolated from squid ink and subsequently functionalized with N‐cadherin antibodies, enabling targeted delivery to endothelial cells that overexpress N‐cadherin. These nanoparticles effectively reverse EndoMT, restore endothelial barrier integrity, and reduce inflammation, ultimately inhibiting an early stage plaque formation^[^ ^99^ ^]^ (**Figure** **6**A).

Recent studies have highlighted miRNA‐145 as a crucial regulator of VSMC phenotype switching, particularly in driving the contractile phenotype and curbing cellular proliferation via targeting of transcription factors such as Klf4, myocardin, and Elk‐1.^[^ ^100^ ^]^ Chin et al. developed CCR2‐targeting amphiphilic peptide micelles (PAMs) for the targeted delivery of miRNA‐145 to atherosclerotic plaques. These micelles effectively suppressed VSMC phenotypic switching and reduced plaque formation and progression.^[^ ^101^ ^]^ Gao et al. investigated the therapeutic potential of transient receptor potential vanilloid subfamily 1 (TRPV1), a thermosensitive cation channel that is activated by capsaicin. In oxLDL‐treated VSMCs, TRPV1 activation induced autophagy, facilitated cholesterol efflux, and reduced foam cell formation. The researchers conjugated CuS nanoparticles with a TRPV1‐specific monoclonal antibody, thus constructing a TRPV1 photothermal switch.^[^ ^102^ ^]^ NIR laser irradiation generated localized temperature increases through the photothermal conversion of CuS, which subsequently opened the TRPV1 channel and allowed for Ca^2+^ influx, leading to autophagy activation. After 12 weeks of treatment, significant reductions in lipid deposition and atherosclerotic lesions were observed in the aortic arches of mouse models (Figure 6B).

Clinical trials such as LEADER and SUSTAIN‐6 have demonstrated that glucagon‐like peptide‐1 (GLP‐1R) agonists significantly reduce MACEs in type 2 diabetes patients. In addition to their glucose‐lowering effects, GLP‐1R agonists may enhance plaque stability by dampening inflammation, restoring endothelial function, and inhibiting VSMC proliferation.^[^ ^103^ ^]^ Maiseyeu et al. developed nanoparticles loaded with the GLP‐1R agonist liraglutide and explored their direct cardiovascular benefits.^[^ ^104^ ^]^ Their findings indicated that low‐dose liraglutide nanoparticles reduced the atherosclerotic burden independently of glucose and weight management, providing compelling evidence for the direct cardiovascular advantages of GLP‐1R agonists.

### 4.2 Nanoparticle‐Based Modulation of Key Pathological Processes

#### 4.2.1 Anti‐Inflammatory Strategies

Encapsulating anti‐inflammatory agents within nanoparticles may help modulate inflammatory responses within atherosclerotic plaques and minimize systemic toxicity. Colchicine, a well‐known anti‐inflammatory agent that inhibits the NF‐κB signaling pathway and the NLRP3 inflammasome, has demonstrated great potential in stabilizing atherosclerotic plaques and reducing plaque instability. Low‐dose colchicine has been shown to prevent cardiovascular events in patients with stable coronary artery disease, but its clinical utility is limited by its narrow therapeutic window and significant adverse effects. To overcome these limitations, Zhu et al. developed HA‐modified Prussian blue nanoparticles (PBNP@HA), which enabled the targeted delivery of colchicine to macrophages within atherosclerotic plaques.^[^ ^105^ ^]^ After four weeks of treatment, col@PBNP@HA significantly attenuated atherosclerotic lesions in a murine model. Similarly, Tang et al. used VHPK peptide‐modified PLGA–PEG nanoparticles for targeted colchicine delivery to inflammatory endothelial cells and achieved superior therapeutic outcomes compared to free colchicine with no notable long‐term safety issues.^[^ ^106^ ^]^

Targeting specific proinflammatory cytokines is another direct approach to mitigating inflammation. IL‐1β is a potent cytokine that has been implicated in atherosclerosis, and clinical studies have suggested that anti‐IL‐1β monoclonal antibodies can effectively prevent adverse cardiovascular events. However, these treatments are also associated with a significant risk of systemic immunosuppression.^[^ ^45^ ^]^ Wu et al. loaded copper‐doped mesoporous silica nanoparticles with IL‐1 receptor antagonists (IL‐1Ra@Cu–MSNs).^[^ ^107^ ^]^ These nanoparticles can target IL‐1 receptors and compete with endogenous IL‐1, leading to potent anti‐inflammatory effects. Additionally, the release of copper ions stimulates ROS production and macrophage apoptosis. IL‐1Ra@Cu–MSNs address the issues of degradation and hepatotoxicity that are associated with the systemic administration of IL‐1Ra and copper ions. In vivo studies indicated that IL‐1Ra@Cu–MSNs significantly reduced intimal thickness, lipid deposition, and macrophage infiltration in murine models.

Upstream inflammatory signaling pathways within macrophages are another promising therapeutic target for atherosclerosis. Platelet‐derived extracellular vesicles, which deliver the NLRP3 inflammasome inhibitor MCC950 have led to significant reductions in plaque size and local inflammatory responses.^[^ ^108^ ^]^ Bai et al. used PEG‐coated superparamagnetic iron oxide nanoparticles (SPIONs) as cores and attached phosphatidylserine‐modified miR‐146a to their surfaces to create miR‐146a‐SPIONs.^[^ ^109^ ^]^ These nanoparticles naturally targeted macrophage scavenger receptors, enabling miR‐146a to inhibit NF‐κB signaling within macrophages. This targeted inhibition attenuated inflammation and promoted plaque stabilization, providing powerful therapeutic benefits while minimizing toxicity.

Li et al. developed a novel metal–organic cage (MOC)‐68‐doped MnO_2_ nanoparticle for the controlled delivery of diallyl trisulfide (DATS), a hydrogen sulfide (H_2_S) donor.^[^ ^110^ ^]^ The proton‐driven conformational flexibility of MOC‐68 allowed for the controlled release of DATS in response to the acidic microenvironment of atherosclerotic plaques. H_2_S, a bioactive molecule, has been shown to inhibit NLRP3 inflammasome activation in foam cells. Additionally, MnO_2_ catalyzed the Fenton reaction with H_2_O_2_, generating oxygen (O_2_), which alleviated hypoxic conditions within the plaque. This process also downregulated HIF‐1 and slowed plaque progression. This dual‐action nanoparticle system offers a promising strategy for addressing inflammation and hypoxia in atherosclerosis (Figure 6C).

#### 4.2.2 Antioxidative Strategies

**Figure 7:**
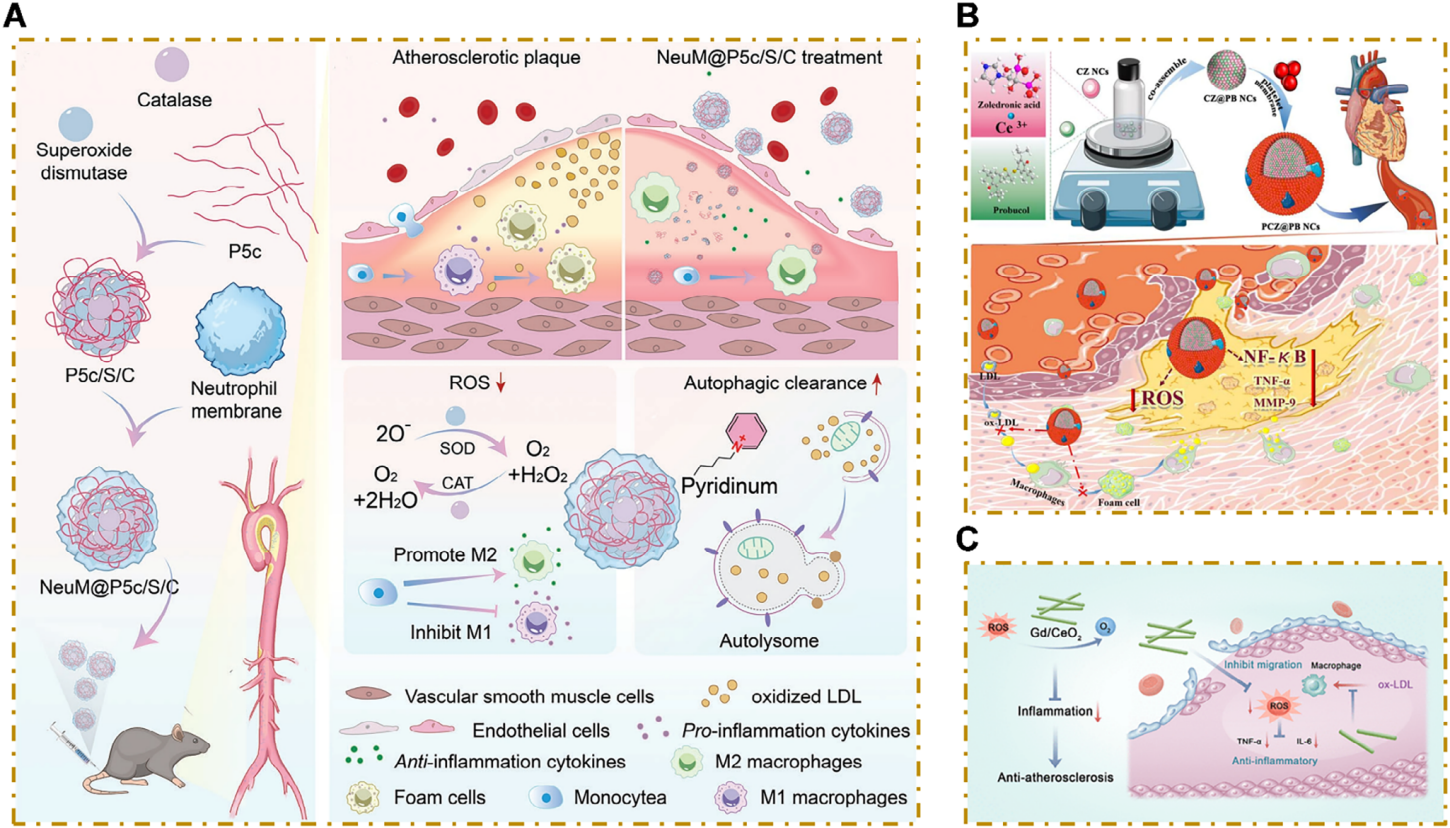
Atherosclerosis nanotherapies which use antioxidant strategies. A) Schematic illustration of P5c with autophagy‐inducing activity for atherosclerosis treatment via codelivery of the antioxidant enzymes superoxide dismutase (SOD) and catalase (CAT). Reproduced with permission.^[^ ^111^ ^]^ Copyright 2025, John Wiley and Sons. B) Preparation and application of the nanozyme (platelet‐membrane‐coated biomimetic nanoplatform, PCZ@PB NCs). Reproduced with permission.^[^ ^113^ ^]^ Copyright 2025, Springer Nature. C) Schematic diagram showing the preparation and mechanism of Gd/CeO_2_ for attenuating atherosclerosis. Reproduced with permission.^[^ ^114^ ^]^ Copyright 2025, American Chemical Society.

Enhancing endogenous antioxidant defenses by delivering antioxidant enzymes is a potent strategy to directly mitigate oxidative stress. SOD catalyzes the conversion of superoxide radicals (O_2_•^−^) into H_2_O_2_ and O_2_, and CAT specifically reduces H_2_O_2_ to H_2_O and O_2_. However, these enzymes cannot permeate cell membranes, which significantly limits their therapeutic potential. To address this, Liang et al. identified the lead material P5c from a library of polypyridiniums and found that it offered substantial intracellular protein delivery efficacy. P5c effectively transported SOD and CAT into the cytoplasm, neutralizing LPS‐induced ROS and promoting macrophage polarization toward the anti‐inflammatory M2 phenotype. P5c also induced autophagy in macrophages and inhibited foam cell formation. By incorporating a neutrophil membrane coating, the NeuM@P5c/S/C nanoparticles showed enhanced inflammation‐targeting specificity and were able to effectively accumulate at the site of atherosclerotic plaques. In mouse models of atherosclerosis, treatment with NeuM@P5c/S/C reduced the size of the necrotic cores and significantly increased collagen content and fibrous cap thickness, thereby promoting plaque stability^[^ ^111^ ^]^ (**Figure** **7**A).

Other innovative strategies involve the use of catalytic nanomaterials known as “nanozymes,” which are designed to mimic the activities of endogenous antioxidant enzymes. For example, Wang et al. synthesized a broad‐spectrum ROS‐eliminating material by covalently conjugating the SOD mimetic agent Tempol and a hydrogen‐peroxide‐eliminating compound of phenylboronic acid pinacol ester onto β‐cyclodextrin. This nanozyme effectively cleared intracellular ROS in macrophages and VSMCs, thus mitigating oxidative stress‐induced inflammation and cellular apoptosis while also stabilizing atherosclerotic plaques.^[^ ^112^ ^]^ Multiple studies have confirmed that cerium dioxide (CeO_2_) nanozymes have excellent SOD and CAT mimic activities. Specifically, Ce^3+^ and Ce^4+^ coexist in cerium oxides, with the charge deficiency of Ce^3+^ balanced by oxygen vacancies that accumulate on the surface (“defects”). Because of their small size, CeO_2_ nanoparticles have a high surface area‐to‐volume ratio. This increased ratio results in an increased number of these surface “defects” and a higher Ce^3+^/Ce^4+^ ratio. The redox cycle between Ce^3+^ and Ce^4+^ enables these nanoparticles to efficiently catalyze the breakdown of superoxide and hydrogen peroxide, leading to a self‐regenerative mechanism that combats ROS accumulation. Fu et al. developed functionalized cerium–zoledronate nanocomposites for the delivery of probucol (PCZ@PB NCs). Platelet‐membrane‐coated PCZ@PB NCs selectively accumulate in atherosclerotic lesions and are efficiently internalized by macrophages, improving ROS clearance and modulating inflammatory responses by downregulating TNF‐α and MMP‐9 expression via NF‐κB inhibition (Figure 7B).^[^ ^113^ ^]^ Further, advances such as gadolinium‐doped CeO_2_ (Gd/CeO_2_) nanozymes have increased the surface Ce^3+^ proportion and enhanced ROS scavenging capabilities. This is particularly beneficial in protecting endothelial cells from oxidative‐stress‐induced damage^[^ ^114^ ^]^ (Figure 7C).

Another promising therapeutic avenue involves targeting NADPH oxidase, which is a major ROS generator in atherosclerosis. Li et al. demonstrated the therapeutic potential of HB‐OLD7 nanoparticles carrying siRNA against *Cybb*, a gene encoding the critical NOX2 subunit of NADPH oxidase.^[^ ^115^ ^]^ The local silencing of *Cybb* expression suppressed neointimal hyperplasia postangioplasty in atherosclerotic rats, highlighting the feasibility of NADPH oxidase inhibition as a nanotherapeutic approach.

In addition to synthetic approaches, numerous studies have explored traditional medicine and natural compounds for their antioxidant and anti‐inflammatory properties. In addition to curcumin, Yu et al. investigated the therapeutic potential of (−)‐epicatechin gallate (ECG), a potent antioxidant that is derived from green tea polyphenols. To overcome ECG's inherent instability, the researchers engineered a dual‐carrier nanoparticle system comprised of acetylated cyclodextrin and HA‐conjugated cyclodextrin. This system enabled targeted ECG delivery to macrophages.^[^ ^116^ ^]^ In the acidic microenvironment of atherosclerotic lesions, the acid‐sensitive acetylated cyclodextrin releases ECG and neutralizes ROS. These actions inhibit macrophage apoptosis and limit VSMC proliferation and migration.

## 5 NDDS Targeting and Controlled Release Mechanisms

Targeting and controlled release strategies enable nanoparticles to selectively accumulate at atherosclerotic lesions and to deliver therapies in a controlled manner, which minimizes off‐target effects and maximizes therapeutic efficacy. This section explores these strategies and highlights their potential applications in atherosclerosis treatment (**Table** **2**).

**Table 2:** Examples of targeted nanoparticles for atherosclerosis.

Year	Name	Carrier/nanoplatform	Cargo	Targeting moieties/target	Animal model	Ref.
Macrophage targeting						
2010	CLIO‐THPC	Cross‐linked dextran‐coated iron oxide (CLIO) nanoparticles	THPC	Crosslinked dextran/polysaccharide receptor	ApoE^−/−^ mice	[58]
2022	HA‐HNSs	CuS/TiO_2_ heterostructure nanosheets	–	HA/CD44	ApoE^−/− ^mice	[59]
2021	HFTNPs	Hyaluronic‐acid‐stabilized Fe(III)–tannic acid nanoparticles	–	HA/CD44	ApoE^−/−^ mice	[60]
2019	AP‐Lipo	Liposome	Pioglitazone	cRGDfK peptide/ανβ3 integrin	ApoE^−/−^ mice	[63]
				Phosphatidylserine (PS)/PS receptor		
2024	9‐CCN‐(125I‐ION/Cur)‐LNPs	125I‐iron oxide nanoparticles	Curcumin	PS and 9‐carboxynonanoate (9‐CCN)/engulfment receptor	New Zealand white rabbits	[65]
2023	IL‐10 mRNA@M‐HNPs	G0‐C14 and PLGA–PEG	IL‐10 mRNA	Mannose/mannose receptors	ApoE^−/−^ mice	[67]
2015	AM NPs	Sugar‐based amphiphilic macromolecules	–	Sugar‐based amphiphilic macromolecules/SR	ApoE^−/−^ mice	[68]
2018	–	Dual‐targeting core–shell nanoplatform	LOX‐1 siRNA	HA/CD44	ApoE^−/−^ mice	[69]
				Apo A‐I/SR		
2024	ASOs@CaP–aSIPRα NPs	Calcium phosphate	mTOR antisense oligonucleotides and anti‐signal‐regulated protein‐α antibody	aSIPRα/SIPR‐α	ApoE^−/−^ mice	[71]
2022	Tr–Arg–PS (TAP) nanomotors	–	Trehalose and l‐arginine	PS/PS receptor	ApoE^−/−^ mice	[72]
2015	PSNP–LXR	PLGA‐*b*‐PEG	GW3965	PS/PS receptor	ApoE^−/−^ mice	[77]
2024	LPLCH	Poly‐ε‐lysine with covalently attached cholesteryl moieties (PLC)	T0901317	HA/CD44	Ldlr^−/−^ mice	[78]
2023	S2PNP‐siEpsin1/2	PLGA	Epsin miRNA	S2P/stabilin‐2	LysM‐DKO‐ABCG1fl/^+^mouse	[88]
2020	S2P‐siCamk2g NPs	PLGA NPs	siCamk2g	S2P/stabilin‐2	Ldlr^−/−^ mice	[93]
2024	Col@PBNP@HA	Prussian Blue nanoparticles	Colchicine	HA/CD44	ApoE^−/−^ mice	[105]
2021	IL‐1Ra@Cu–MSNs	Mesoporous silica nanoparticles	IL‐1Ra and Cu^2+^	IL‐1Ra/IL‐1 receptor	ApoE^−/−^ mice	[107]
2022	miR‐146a‐SPION	Superparamagnetic iron oxide NP	miR‐146a	Spherical nucleic acid/SR	ApoE^−/−^ mice	[109]
Endothelial cell targeting						
2023	BUD–l‐Arg@PSA	Polysialic acid	Budesonide and l‐arginine	polysialic acid (PSA)/E‐selectin	ApoE^−/−^ mice	[95]
2021	Psel‐lipo/shRAGE lipoplexes	Cationic PEGylated liposomes	RAGE shRNA	P‐selectin binding peptide/P‐selectin	ApoE^−/−^ mice	[96]
2024	MNP@PA/BSA@NcadAb	Melanin nanoparticles	N‐cadherin Ab	N‐cadherin Ab/N‐cadherin	–	[99]
2023	VHPK–PLGA@COL	PLGA–PEG–COOH	Colchicine	VHPK peptide/VCAM‐1	ApoE^−/−^ mice	[106]
2015	VHPK–CCL–anti‐miR‐712	Coated, cationic lipoparticles (CCLs)	Anti‐miR‐712	VHPK peptide/VCAM‐1	ApoE^−/−^ mice	[117]
2023	Low molecular weight heparin–unsaturated fatty acid (LMWH–uFA)	Low molecular weight heparin	Unsaturated fatty acid (uFA) and rapamycin (RAP)	Low molecular weight heparin/P‐selectin	ApoE^−/−^ mice	[118]
2006	–	Paramagnetic perfluorocarbon nanoparticles	Fumagillin	Arg–Gly–Asp (RGD) mimetic/αvβ3 integrin	New Zealand white rabbits	[119]
VSMC targeting						
2021	miR‐145 micelle	Peptide amphiphile micelle	miR‐145	MCP‐1 peptide/CCR2	ApoE^−/−^ mice	[101]
2018	CuS–TRPV1	CuS nanoparticles	–	TRPV1‐specific monoclonal antibody/TRPV1	ApoE^−/−^ mice	[102]
Biomimetic nanoparticles						
2024	Curc@Lignin@TA@PL	Lignin‐based nanoparticles	Curcumin	Platelet membrane coated	–	[64]
2024	MM@DA–pCD@MTX	Poly(isobutene‐*alt*‐maleic anhydride) (PIAMA)	β‐Cyclodextrin (β‐CD) and methotrexate (MTX) and dopamine (DA)	Macrophage membrane coated	ApoE^−/−^ mice	[85]
2021	MCC950‐PEVs	Platelet‐derived extracellular vesicles	MCC950	Platelet‐derived extracellular vesicles	ApoE^−/−^ mice	[108]
2024	NeuM@P5c/S/C	P5c	SOD and CAT	Neutrophil‐membrane‐coated	ApoE^−/−^ mice	[111]
2022	PCZ@PB NCs	Ceria‐ZOL nanocomposites	Probucol	Platelet‐membrane‐coated	ApoE^−/−^ mice	[113]
Stimuli‐responsive nanoparticles						
2023	PLCDP@PMH	Poly(2‐methylthio ethanol methacrylate), PMEMA	T0901317 and prednisolone and β‐cyclodextrin	ROS/MMP dual‐responsive	ApoE^−/−^ mice	[83]
				HA/CD44 receptor		
2020	RAAM NP	Acetalated α‐cyclodextrin	miR‐33	pH responsive	ApoE^−/−^ mice	[87]
				cRGDfK peptide/αvβ3 integrin		
2024	CAR‐M/β‐CD LNP	Anti‐CD47 chimeric antigen receptor (CAR) macrophage	Hydroxypropyl 𝛽‐cyclodextrin	ROS‐responsive	–	[94]
				anti‐CD47 chimeric antigen receptor/CD47		
2024	DMM	Metal–organic cage (MOC)‐68‐doped MnO_2_ nanoparticles	Diallyl trisulfide (DATS)	pH responsive	ApoE^−/−^ mice	[110]
2024	ECG‐NPs	Acetylated cyclodextrin and hyaluronic‐acid‐conjugated cyclodextrin	(−)‐epicatechin gallate (ECG)	pH responsive	ApoE^−/−^ mice	[116]
				Hyaluronic acid‐conjugated cyclodextrin/CD44 receptor		
2022	HR_RAP_ NPs	pH sensitive hyaluronic acid nanoparticles	All‐*trans* retinal and rapamycin	pH responsive	ApoE^−/−^ mice	[122]
2022	TPTS/C/T	DSPE–PEG–CREKA	α‐tocopherol polyethylene glycol derivative and simvastatin and ticagrelor	ROS responsive	ApoE^−/−^ mice	[123]
				CREKA peptide/fibrin		
2021	Simvastatin acid (SA) PAM@red blood cells (RBCs)	Cationic poly(amidoamine) (PAMAM) dendrimers	Simvastatin acid	ROS and shear stress responsive	ApoE^−/−^ mice	[124]
2022	SIM@HA‐MSN	Mesoporous silica nanoparticles	Simvastatin	Hyaluronidase (HAase)‐responsive	C57BL/6 mice	[125]
				HA/CD44 receptor		
2022	T/R NPs	PLGA–Pep–PEG	Rapamycin	Cathepsin K (CTSK) responsive	ApoE^−/−^ mice	[126]
				c(RGDfC) peptide/αvβ3 integrin		

### 5.1 Cellular Targeting Strategies

NDDS can achieve precise targeting by incorporating specific ligands or molecules that selectively bind to surface markers on target cells. VCAM‐1 and P‐selectin are key adhesion molecules that are upregulated on activated endothelium in inflamed plaques and facilitate inflammatory cell adhesion and transmigration. Harnessing VCAM‐1‐binding peptides is a common method for targeting the inflamed endothelium. Kheirolomoom et al. developed coated cationic lipoparticles (CCLs) for oligonucleotide‐based therapeutics, such as anti‐miRNA, to target atherosclerotic plaques.^[^ ^117^ ^]^ These CCLs are functionalized with the VHPK peptide, which selectively binds to VCAM‐1. The neutral lipid coating enhances transfection efficiency and reduces toxicity, making them ideal for genetic delivery to atherosclerotic lesions. When loaded with anti‐miR‐712, these nanoparticles effectively downregulate the miR‐712 expression, which is induced by disturbed flow, and restore expression of the target gene – tissue inhibitor of metalloproteinase 3 —thereby inhibiting metalloproteinase activity and improving plaque stability.

Fucoidan, sulfated glucans, and heparin are natural ligands that bind to P‐selectin. Wang et al. engineered a low molecular weight heparin‐unsaturated fatty acid conjugate (LMWH–uFA) that self‐assembles to coat the anti‐inflammatory drug rapamycin.^[^ ^118^ ^]^ Low molecular weight heparin guides the nanoparticles to the lesion and competes with monocytes and platelets for P‐selectin, thus reducing inflammation and thrombotic development. The unsaturated fatty acids and rapamycin, which are loaded into the LMWH–uFA platform, also have lipid‐regulating and anti‐inflammatory effects, which significantly reduced plaque areas in mouse models.

αvβ3 integrin is upregulated in atherosclerotic plaques, particularly within neovasculature and activated endothelial cells, and especially in response to proangiogenic growth factors. Plaque angiogenesis accelerates plaque progression by enabling the infiltration of inflammatory macrophages and lipid deposition, which contributes to plaque growth and destabilization. The increased vulnerability of the neovasculature also heightens the risk of intraplaque hemorrhage and plaque rupture. Winter et al. used αvβ3 integrin‐targeted paramagnetic nanoparticles to deliver the antiangiogenic agent fumagillin in hyperlipidemic New Zealand white rabbits.^[^ ^119^ ^]^ These nanoparticles enabled noninvasive molecular imaging of angiogenesis within plaques, allowing for the visualization and quantification of plaque neovascularization. Fumagillin, delivered via αvβ3 integrin targeting, reduced neovascular density in the adventitia. This targeted delivery strategy allowed for a significant reduction in the required dose of fumagillin, minimizing potential dose‐dependent adverse effects.

Macrophages are central players in atherosclerosis. Their activation has been associated with the overexpression of numerous receptors, which may serve as targets for nanoparticle‐based therapies. Notable examples of these receptors include the HA receptor CD44, scavenger receptors, mannose receptors, and folate receptors. Stabilin‐2 is a multifunctional receptor for HA, heparin, and phosphatidylserine that is expressed on macrophages and endothelial cells within atherosclerotic plaques. Nanoparticles carrying the S2P peptide can bind to Stabilin‐2, facilitating selective targeting.^[^ ^120^ ^]^ Similarly, phosphatidylserine on the surface of apoptotic macrophage‐derived foam cells can direct nanoparticles to atherosclerotic plaques. Li et al. developed a CT/SPECT nanoprobe by conjugating the radionuclide ^99m^Tc and the phosphatidylserine‐targeting molecule Annexin V to gold nanoparticles coated with amino polyethylene glycol.^[^ ^121^ ^]^ This nanoprobe specifically targeted apoptotic foam cells, aiding in the detection of vulnerable plaques.

### 5.2 Stimuli‐Responsive Nanoparticles

The atherosclerotic microenvironment is characterized by several distinctive features, including pH changes, oxidative stress, altered hemodynamics, and the presence of specific enzymes. These unique pathophysiological conditions provide opportunities to design nanoparticles that can respond to these stimuli, enabling controlled and targeted drug release within atherosclerotic lesions. As previously mentioned, the inflammatory nature of atherosclerosis leads to a slight acidification of the environment. To leverage this property, some research groups have designed pH‐sensitive nanoparticles that incorporate chemical bonds that selectively break or undergo structural modification under mildly acidic conditions, enabling targeted drug release within atherosclerotic plaques. For example, Cheraga et al. developed pH‐responsive nanoparticles by conjugating all‐*trans* retinoic acid (ATRA) to HA via a hydrazone bond and encapsulating rapamycin within the coating.^[^ ^122^ ^]^ These nanoparticles can target plaques through HA–CD44 interactions. In the acidic inflammatory microenvironment, the hydrazone bond is hydrolyzed and releases both ATRA and rapamycin.

High ROS levels in atherosclerotic plaques can also serve as a trigger for drug release. Zhao et al. conjugated the pharmacophore of simvastatin to an α‐tocopherol polyethylene glycol derivative via a ROS‐responsive thioketal bond, forming a ROS‐responsive simvastatin nanoprodrug (TPTS).^[^ ^123^ ^]^ TPTS was then encapsulated within a fibronectin‐targeting CREKA peptide and ticagrelor, forming TPTS/C/T. Fibronectin, which is common in atherosclerotic plaques, enables the TPTS/C/T complex to localize specifically to the plaques. In response to the increased ROS levels within the plaques, the ROS‐sensitive bonds in the nanoparticles are cleaved, releasing thiolated simvastatin, which is then hydrolyzed into its active form. Codelivery of ticagrelor and α‐tocopherol with simvastatin enhances the therapy's overall antiatherosclerotic effects.

As atherosclerotic plaques progress, they become increasingly involved in hemodynamic regulation. Under normal conditions, arterial wall shear stress levels range from 1 to 6 Pa in humans, but in plaque‐obstructed regions, shear stress can abruptly rise to 100 Pa because of increased blood flow velocity and turbulence. Elevated shear stress can further exacerbate endothelial cell damage, promote vascular inflammation, and perpetuate the progression of atherosclerosis. Some sophisticated nanoparticle designs have transformed these changes into an opportunity for targeted drug delivery. Cationic poly(amidoamine) (PAMAM) dendrimers have a highly branched structure with excellent water solubility, tunability, and functional ease. However, their positively charged surface amino groups hinder their clinical applications. Shen et al. addressed these challenges by neutralizing their positive surface charges with negatively charged simvastatin acid (SA), leading to the formation of cross‐linked dendrimer nanoparticles (SA PAM). SA PAM can adhere to the negatively charged surface of red blood cells (SA PAM@RBCs) and be transported to atherosclerotic plaques via blood flow.^[^ ^124^ ^]^ The high degree of shear stress at the plaque site causes the SA PAM to detach, facilitating SA accumulation within the lesion.

Certain enzymes, including hyaluronidase (HAase), MMPs, and cathepsin K (CTSK), are upregulated in the atherosclerotic microenvironment. By linking these enzymes to specific substrates, enzyme‐sensitive drug release can be achieved. Song et al., for example, developed a HAase‐responsive, macrophage‐targeted drug delivery system by loading MSNs with simvastatin and encapsulating them in a HA coating. The resultant platform was termed “SIM@HA–MSN.”^[^ ^125^ ^]^ SIM@HA–MSN achieved high loading and enzyme‐responsive release and demonstrated strong anti‐inflammatory and antifoaming effects in vitro with relatively low cytotoxicity. Similarly, Fang et al. synthesized a CTSK‐sensitive polymer, PLGA–Pep–PEG, and self‐assembled it with an integrin αvβ3‐targeting polymer, forming nanoparticles (T/R NPs) with targeting capabilities and CTSK responsiveness.^[^ ^126^ ^]^ T/R NPs encapsulating rapamycin were shown to effectively accumulate in plaques and inhibit atherosclerotic progression. Muñoz‐Hernando et al. aimed to use endogenous retention mechanisms to selectively retain nanoparticles within atherosclerotic plaques. Sphingomyelinase, which is secreted by endothelial cells and macrophages, converts membrane‐bound sphingomyelin to ceramides.^[^ ^127^ ^]^ Ceramides’ hydrophobic interactions promote LDL aggregation and retention in arterial walls.^[^ ^128^ ^]^ Researchers stabilized hydrophobic iron oxide nanoparticles with sphingomyelin, forming sphingomyelin iron oxide nano micelles (SPHIONMs).^[^ ^129^ ^]^ In atherosclerotic plaques, sphingomyelinase disrupts the colloidal stability of SPHIONMs, leading to aggregation. This process mimics the LDL retention mechanism in the subendothelial space and enhances SPHIONM accumulation at the lesion.

## 6 Conclusion and Outlook

Atherosclerosis remains a major global health concern. Although conventional treatment strategies—including lifestyle modifications, pharmacological interventions, and surgical procedures—have demonstrated some efficacy, they are often constrained by their limited ability to address the underlying causes and multifaceted pathophysiology of the disease. In this context, NDDS has emerged as a promising therapeutic platform, offering the potential to transform the treatment landscape of atherosclerosis through targeted, efficient, and personalized drug delivery.

Given the complex pathogenesis underlying atherosclerosis, previous researchers have developed diverse strategies to design, test, and implement NDDS. One key approach involves targeting disease‐specific biomarkers that are expressed by the macrophages, endothelial cells, and VSMCs that lie within atherosclerotic plaques, which enables more precise drug delivery. Nanoparticles can also be engineered to respond to unique features of atherosclerotic lesions, including elevated ROS levels and acidic pH, ensuring site‐specific drug release. This targeted approach enhances therapeutic efficacy and minimizes systemic toxicity. To further improve biocompatibility and extend nanoparticle half‐lives, natural polymers (e.g., hyaluronic acid, albumin) and PEG are commonly used for surface modification. These modifications enhance nanoparticle stability, prolong circulation time, and increase the likelihood of effective targeting.

Despite promising results from preclinical studies, translating these therapeutic strategies into clinical settings remains a potent challenge.^[^ ^130^ ^]^ Future research should focus on the following key areas. 1) Enhancing targeting efficiency—despite improved accumulation of therapeutic agents at target sites compared to conventional therapies, the overall targeting efficiency of current NDDS platforms remains suboptimal. This inefficiency compromises therapeutic outcomes and raises the risk of systemic toxicity due to off‐target distribution. Future efforts should focus on improving targeting precision. This could be accomplished through the screening and optimization of novel targeting ligands (e.g., peptides, antibodies, aptamers), the engineering of biomimetic or cell‐membrane‐coated nanoparticles to exploit endogenous homing mechanisms, and/or the implementation of spatiotemporally staged delivery systems. 2) Biosafety evaluation—one of the major barriers to the clinical application of NDDS is an incomplete understanding of their in vivo behavior, particularly related to pharmacokinetics, biodistribution, biodegradability, and long‐term safety. A major concern is the potential for chronic inflammation, fibrosis, or immune dysregulation arising from prolonged retention of non‐degradable or slowly degradable nanoparticles in plaques or off‐target organs such as the liver, spleen, and kidneys. Current biosafety assessments have predominantly relied on in vitro cytotoxicity assays and short‐term animal studies, which fail to capture the complexity of nanoparticle‐host interactions in vivo. Moreover, substantial interspecies differences—such as in lipid metabolism, immune responses, and plaque biology—limit the extrapolation of preclinical data to human patients. Thus, future research should aim to elucidate the metabolic and clearance pathways nanocarriers, develop more fully biodegradable and nonimmunogenic materials, and establish long‐term in vivo models that better mimic human pathophysiology. Once preclinical safety and efficacy profiles have been validated, well‐designed, multicenter clinical trials be undertaken to evaluate improvements in clinically relevant endpoints, such as reductions in MACEs. 3) Investigation of mechanisms underlying nanoparticle actions—although most current studies have emphasized the phenotypic or functional outcomes of nanoparticle therapies, the molecular mechanisms underpinning their therapeutic effects remain poorly characterized. Understanding how nanoparticles interact with target cells, modulate signaling pathways, alter local microenvironments, and/or influence immune–metabolic crosstalk is essential to refine nanocarrier design and improve therapeutic precision. Advanced analytical techniques should be used to investigate these pathways and refine therapeutic approaches. 4) Standardization and optimization of manufacturing processes—because of the intricate components and preparatory procedures required to produce many nanoparticles, conventional laboratory‐scale purification methods and large‐scale industrial manufacturing of NDDS have remained out of reach. Incorporating advanced manufacturing technologies, such as microfluidics and automated production systems, will facilitate the production of nanoparticles with consistent sizes, drug‐loading efficiencies, and physicochemical properties. Further, establishing rigorous quality control measures and standardized regulatory guidelines is essential to supporting large‐scale NDDS production. 5) Development of personalized and multifunctional therapeutic platforms—atherosclerosis is a highly heterogeneous and systemic disease. It is also influenced by patient‐specific factors such as genetics, metabolic states, immune profiles, and disease stage. This necessitates the development of personalized therapies. Integrating high‐throughput screening technologies, system biology approaches, and artificial intelligence could support the rational design of tailored nanocarriers. In addition, multifunctional nanoplatforms capable of simultaneously delivering multiple therapeutic agents—or combining diagnostic, therapeutic, and real‐time monitoring capabilities—hold promise for comprehensive and adaptive disease management. For example, codelivery systems that target inflammation, oxidative stress, and lipid metabolism concurrently may yield synergistic therapeutic benefits. 6) Interdisciplinary collaborations—the successful advancement of nanoparticle‐based atherosclerosis therapy will require close collaboration across multiple scientific and industrial disciplines, including chemistry, materials science, biology, pharmacology, and clinical medicine. Strengthening interdisciplinary partnerships will accelerate the resolution of continued technical challenges.

In conclusion, NDDS represents a promising treatment for atherosclerosis. With continued technological advancements and clinical research, nanomedicine could provide more precise, effective, and personalized treatments for this disease, driving a transformative shift in cardiovascular disease therapy.

## Conflict of Interest

The authors declare no conflict of interest.

## Author Contributions

Y.Z., F.L., and L.L. contributed equally to this work and shared the first authorship. J.T., C.L., and X.S. conceptualized and structured the paper. Y.Z., F.L., and L.L. wrote the initial draft. Z.A. and H.Z. prepared the figures and tables. H.L., R.M., R.Z., and M.Z. performed the literature review. Q.D. and C.L. revised and edited the paper. Y.L. performed supervision.
